# Structural basis for DNA recognition by a viral genome-packaging machine

**DOI:** 10.1073/pnas.2406138121

**Published:** 2024-08-08

**Authors:** Maria Chechik, Sandra J. Greive, Alfred A. Antson, Huw T. Jenkins

**Affiliations:** ^a^York Structural Biology Laboratory, Department of Chemistry, University of York, York YO10 5DD, United Kingdom; ^b^York Biomedical Research Institute, University of York, York YO10 5NG, United Kingdom

**Keywords:** bacteriophage HK97, DNA packaging, virus assembly

## Abstract

DNA recognition is critical for assembly of double-stranded DNA viruses, particularly for the initiation of packaging the viral genome into the capsid. The key component that recognizes viral DNA is the small terminase protein. Despite prior studies, the molecular mechanism for DNA recognition remained elusive. Here, we address this question by identifying the minimal site in the bacteriophage HK97 genome specifically recognized by the small terminase and determining the structure of this complex by cryoEM. The circular small terminase employs an entirely unexpected mechanism in which DNA transits through the central tunnel, and sequence-specific recognition takes place as it emerges. This recognition stems from a substructure formed by the N- and C-terminal segments of two adjacent protomers which are unstructured when DNA is absent. Such interaction ensures continuous engagement of the small terminase with DNA, enabling it to slide along the DNA while simultaneously monitoring its sequence. This mechanism allows locating and instigating packaging initiation and termination precisely at the specific *cos* sequence.

## Significance

The most abundant group of viruses, comprising tailed bacteriophages and evolutionarily related herpesviruses, specifically recognize their genomic DNA and package it into a preformed shell (capsid). The key protein involved in DNA recognition is the small terminase. Whether the small terminase recognizes DNA by wrapping it around itself or binding inside the internal channel remained the subject of major debate in virology. We identified the specific DNA sequence required for recognition and determined its structure in complex with the small terminase from *Escherichia coli* bacteriophage HK97 using cryoEM. The structure suggests a model where the small terminase can slide along DNA while surveying its sequence to identify and bind the specific site.

The large group of double-stranded DNA (dsDNA) viruses comprising tailed bacteriophages and herpesviruses assemble by packaging their DNA into preformed procapsids (1–3). The key component ensuring specific recognition of viral DNA from the mixture of all the nucleic acids contained in the host cell, and initiating its packaging is the small terminase protein (4). Packaging initiation occurs when the small terminase binds to a specific sequence (*pac* or *cos*) in the genomic DNA, facilitating the cleavage of the dsDNA by large terminase and subsequent assembly of the large terminase bound DNA complex onto the portal protein vertex of the capsid for packaging to begin (1, 2, 5). Insertion of a complete copy of the genome into the capsid is followed by packaging termination, resulting in cleavage of the DNA by the nuclease domain of large terminase. Unlike headful (*pac*) packaging, in which cleavage occurs randomly after approximately 102 to 105% of viral genome has been packaged, in *cos* viruses, such as λ or HK97, termination cleavage occurs precisely at the boundary of the next genomic copy, defined by the *cos* sequence, and requires small terminase (3, 6). After termination cleavage, the large terminase motor:DNA complex is transferred to a new empty capsid where it continues packaging the next copy of the genome in the concatemeric DNA (7–9).

The mechanism of DNA recognition by small terminase remains the subject of a major debate despite the availability of structures of small terminases from more than 10 dsDNA bacteriophages [*SI Appendix*, Fig. S1 (6, 10–24)] solved in the absence of DNA. All small terminase structures broadly resemble each other in architecture, with 8 to 12 protomers oligomerized in a ring to form a central channel in the middle, with the N-terminal segments arrayed around the outside. The C-terminal region is often disordered (6, 13, 14, 17, 19). While in many small terminase structures the N-terminal segment has the helix–turn–helix (HTH)-like fold presumed to constitute the DNA-binding domain (DBD), and sufficient for DNA binding in λ phage and SF6 (10, 12, 25), in some cases, for example, P22, 44RR, and HK97, no well defined DBD was observed. Such structural variation has confounded development of a universal consensus for the mechanism of small terminase binding to DNA. However, based on the available data, two alternative models have been proposed: a wrapping model where the DNA wraps around the outside of the circular oligomer by interacting with the N-terminal DBDs (11, 19–21, 25) and a threading model where the DNA is threaded through the internal channel of the circular oligomer (18, 20). However, despite being the focus of research for many years, no structure of a DNA-bound small terminase complex has been determined.

*Escherichia coli* bacteriophage HK97 has been the subject of intensive research, and along with λ and P22 (18, 25, 26), serves as an excellent model system for understanding the mechanism of virus assembly and DNA packaging. Recent work on HK97 developed an in vitro packaging system and determined the structures for both large and small terminases (6, 27). In this work, we identified the boundaries of the minimal sequence within the *cos* region that is specifically recognized by the small terminase and determined the structure of this complex by electron cryomicroscopy (cryoEM) revealing the molecular mechanism for recognition of the specific *cos* sequence.

## Results

### Specific Small Terminase Binding Site within the HK97 *cos* Region.

**Fig. 1.**
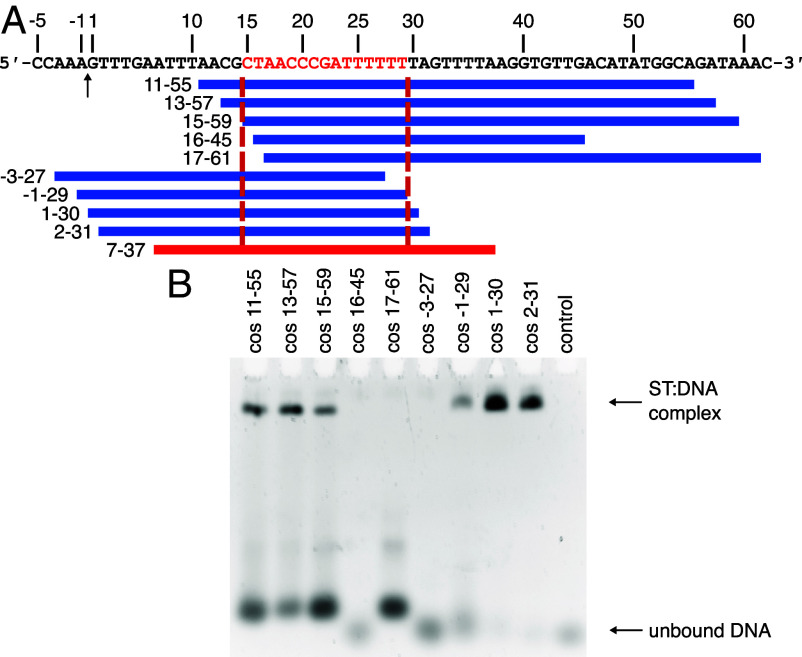
DNA binding site of small terminase. (*A*) Sequence and schematic diagram of HK97 *cos* site: the arrow indicates cleavage site (between position −1 and 1) during genome packaging. Red segment (15 to 29)—essential DNA binding site. Blue blocks represent different dsDNA fragments. Red block (7 to 37)—oligonucleotide that was used for complex formation. Red dotted lines show the border of DNA binding site. (*B*) EMSA of small terminase (ST) with dsDNA. Control lane—no protein. The minor additional band in lanes 1 to 3 and 5 is most likely a different protein:DNA complex resulting from copurification of an *E. coli* DNA-binding protein with small terminase.

Preliminary data indicated that the recognition site for small terminase lies between positions −80 and +472 spanning the cleavage site (between −1 and +1) in the *cos* region (28). To define the binding site more precisely, we used progressively shorter dsDNA oligonucleotides encompassing the *cos* cleavage site to probe for small terminase:DNA binding by electrophoresis mobility shift assays (EMSA) using fluorescently labeled DNA (*SI Appendix*, Fig. S2). Subsequently, unlabeled DNA oligonucleotides were used to locate the boundaries of the binding site with single base pair resolution (Fig. 1). The minimal region necessary for small terminase binding comprises the 15 bp (position 15 to 29) starting from 15 bp downstream of the *cos* cleavage site.

### CryoEM Structure of the Small Terminase:DNA Complex.

**Fig. 2.**
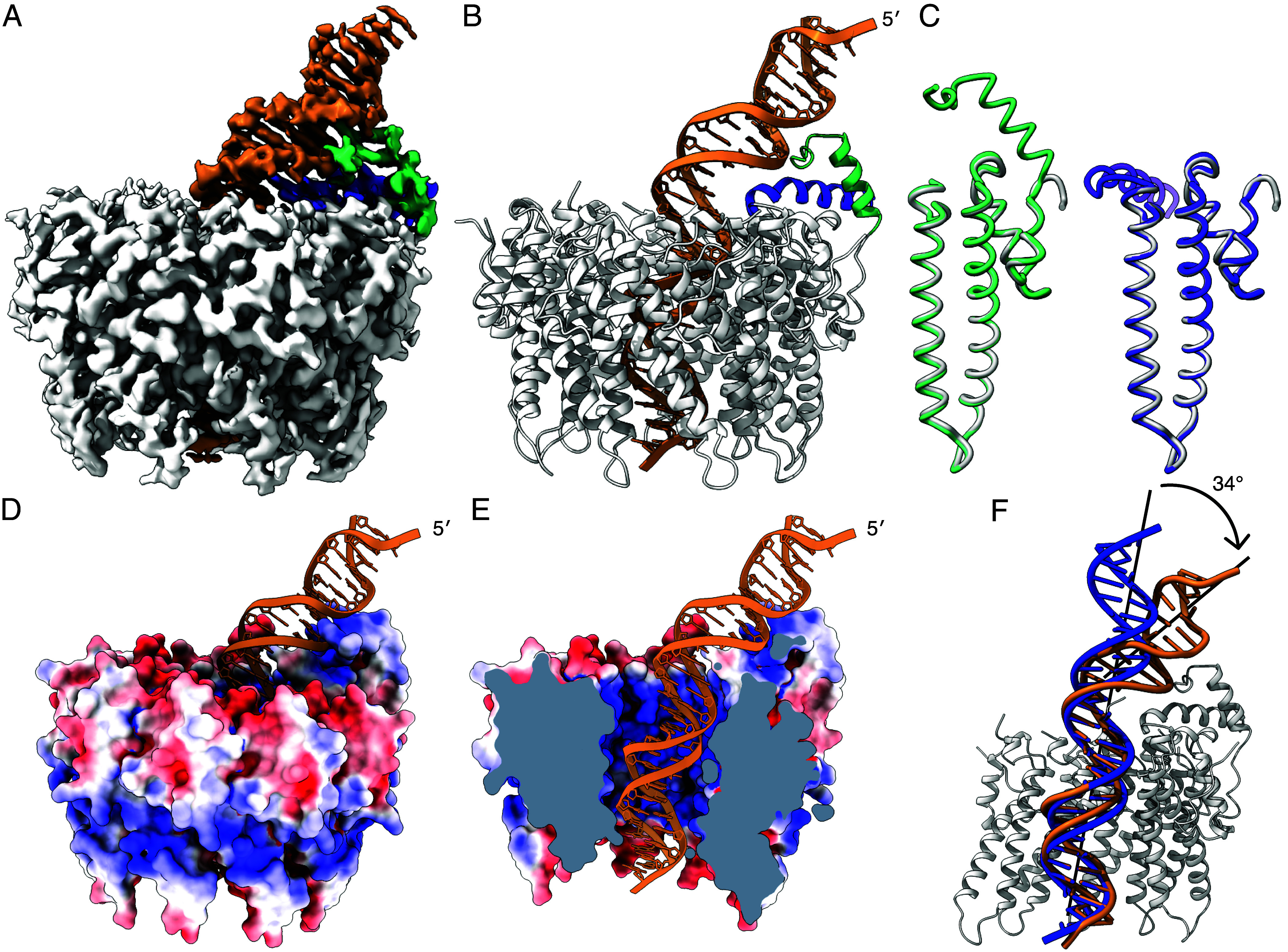
Structure of small terminase:DNA complex. (*A*) CryoEM map and (*B*) ribbon representation of small terminase:DNA complex. Nine protomers (gray) form the nonameric structure of small terminase. N-terminal helix from chain A (green) and a C-terminal helix from chain B (purple) form a DNA-binding substructure that directly interacts with DNA (gold). (*C*) Superposition of one monomer (gray) from the previously determined X-ray structure of small terminase (PDB 6z6e) with chain A (green) and B (purple) from the cryoEM structure. (*D*) Molecular surface of the small terminase colored according to electrostatic charge, with DNA shown as ribbon. Negative and positive charges are in red and blue respectively, varying from −5 to +5 kT/e. (*E*) Cross-section of the map showing the electrostatic surface charges (calculated as in *D* inside the channel). (*F*) Alignment of DNA (gold) with ideal B-form DNA of the same sequence (blue). Mainchain alignment of 11 base pairs from the bottom end of the DNA was performed with LSQ in Coot.

EMSA confirmed that dsDNA fragments of several different lengths: 21, 25, and 31 bp, all containing the minimal site in the center of the fragment, were sufficient to enable formation of a complex with small terminase (*SI Appendix*, Fig. S3). The complex of small terminase with the longest of these fragments was purified from unbound DNA using size exclusion chromatography (*SI Appendix*, Fig. S4) and the structure of this complex was determined at a resolution of 3.0 Å by cryoEM (Fig. 2 and *SI Appendix*, Figs. S5–S9 and Tables S1 and S2).

In the complex, DNA is threaded through the central channel, bent toward one side of the small terminase nonamer to form an asymmetric complex. As observed in the previously reported crystal structure of small terminase in the absence of DNA (6) the oligomer contains nine protomers surrounding a central channel. In the crystal structure, the diameter of the channel at its narrowest point is 18 Å while in the cryoEM structure, the channel is ~3% larger. While part of this difference in size may reflect the accuracy of the calibrated pixel size for the cryoEM structure, it is possible that the channel expands slightly to accommodate DNA. In the structure of the small terminase:DNA complex residues Asp24 to Asp124 are well defined for all nine protomers (Fig. 2 *A* and *B*). The mainchain of this region in the cryoEM structure aligns to the crystal structure with a rmsd of 0.5 Å (Fig. 2*C*) indicating that binding to DNA does not change the conformation of the oligomerization region of small terminase. Strikingly, in the small terminase:DNA complex, density for the N-terminal residues Asp3-Val23 of one protomer and the C terminus—residues Gly125 to Asp145—of the adjacent protomer is clearly resolved in the map. The N terminus of one protomer and the C terminus of the adjacent protomer each form an α-helix that pack against each other, in a manner reminiscent to that in a classical HTH motif, to create a DNA-binding substructure. The orientation of these two helices is stabilized by 6 interhelix hydrogen bonds (*SI Appendix*, Fig. S10) and the buried surface area between the helices is 430 Å^2^. These helices form a scaffold that places the N-terminal arm (NTA) of the N-terminal helix in the minor groove, while the end of the C-terminal helix is inserted into the major groove where the DNA is bent as it exits the central channel (Fig. 2).

**Fig. 3.**
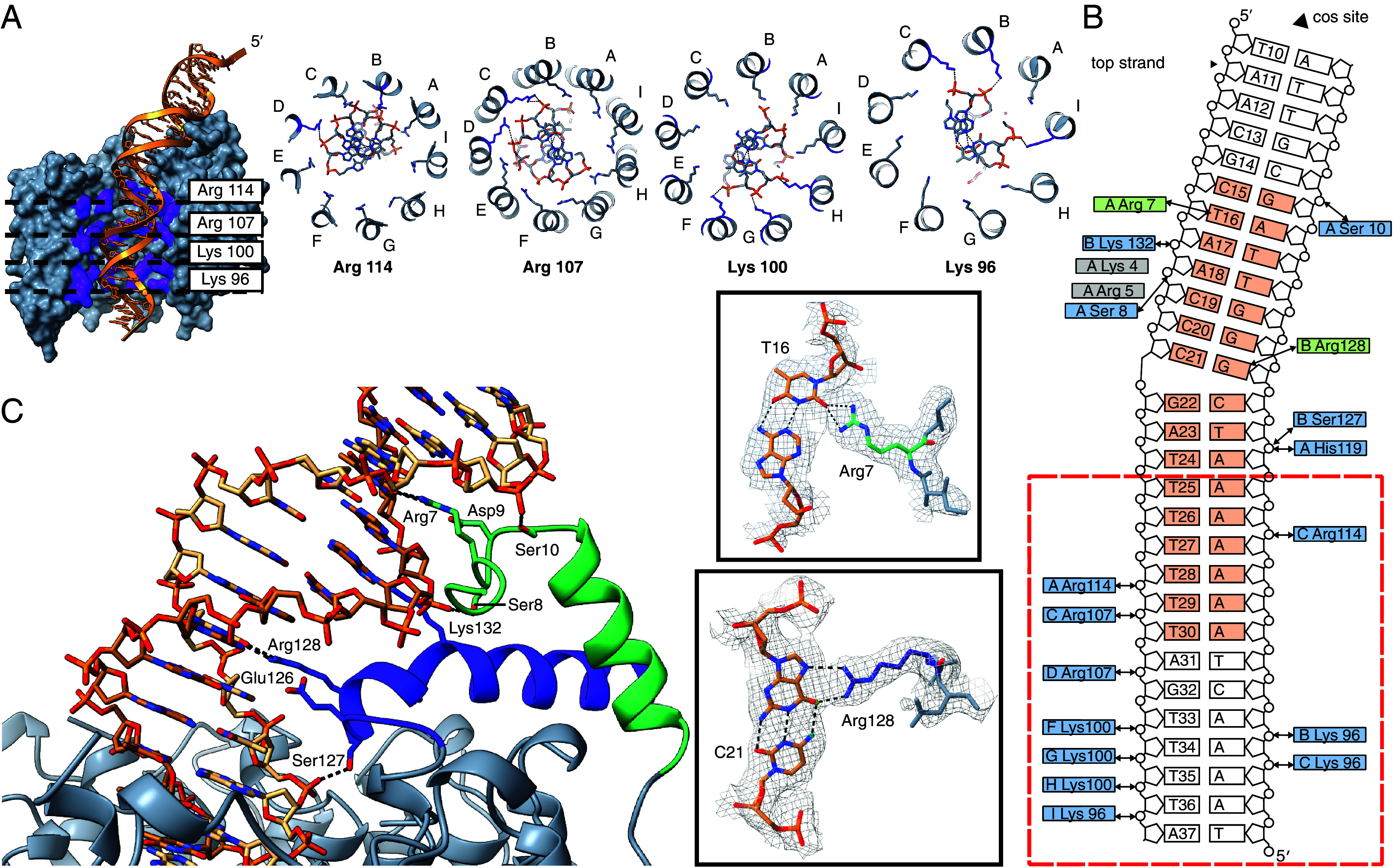
Interaction of small terminase with dsDNA. (*A*) Cross-section of small terminase (gray) showing four positively charged rings formed by arginine and lysine residues (purple) with DNA depicted in gold. Protomers are labeled with letters A to I. (*B*) Schematic representation of overall small terminase: DNA interaction. The *cos* cleavage site is positioned 10 nucleotides upstream of the DNA shown. Residues forming specific interactions with DNA are shown in green and residues forming hydrogen bonds with the backbone phosphates of DNA are shown in blue. The interaction site on DNA for residues shown in gray was not unambiguously defined in the cryoEM density. The minimal small terminase binding site is shown in orange. Red dashed line outlines the position of the small terminase channel. Black arrows indicate hydrogen bonds. (*C*) The specific DNA-binding substructure. CryoEM density along with corresponding models of Arg7:T16 and Arg128:G at position 21 are shown inside boxes. Hydrogen bonds are shown by black dashed lines.

Although a 31 bp oligonucleotide (7 to 37 of the *cos* sequence) was used to prepare the complex for cryoEM, only 28 base pairs are clearly resolved in the cryoEM density. The density for base pairs inside the channel is better resolved with density for nucleotides becoming less well defined as the DNA extends away from small terminase. Importantly, it was possible to unambiguously assign each nucleotide in the DNA within the channel. This enabled us to define the orientation of the dsDNA within the complex: the less well-ordered end that extends beyond the protein is the portion closest to the *cos* cleavage site with the small terminase binding site bound partially within the tunnel (Fig. 3*A*) and partially outside the nonamer by the DNA-binding substructure (Fig. 3*B*).

### Interactions with DNA Bound in the Central Channel.

The internal surface of the channel of small terminase is positively charged (Figs. 2*E* and 3*A*). Four rings of lysine and arginine residues: Lys96, Lys100, Arg107, and Arg114 line the channel and form hydrogen bonds with phosphates of the DNA backbone (Fig. 3*A*). The DNA duplex forms hydrogen bonds with 2 to 3 residues from adjacent protomers at each “level”, as it threads through the central channel. The composition of the group of protomers that contact the DNA at each level rotate around the nonamer with respect to the DNA-binding substructure. For example, the DNA forms bonds with Lys96 (from three protomers B, C, and I), followed by Lys100 (from three adjacent protomers F, G, and H) deeper in the tunnel, then Arg107 from two adjacent protomers (C and D) and finally Arg114 from two protomers (A and C) adjacent to the tunnel entrance closest to the DNA-binding substructure formed from protomers A and B. His119 and Ser127 from protomers A and B, respectively, located at the top of the channel additionally form hydrogen bonds with the phosphates of the DNA (Fig. 3*B*). As DNA threads through the central channel, the rings of charged residues lining the channel interact with the DNA, which has a deformed structure when compared to the canonical B-form. This distortion results in narrowing of the minor groove and widening of the major groove (*SI Appendix*, Fig. S11) and coincides with the A-tract in the sequence of the minimal specific binding site.

### Specific Protein–DNA Interactions Formed by the DNA-Binding Substructure.

Direct hydrogen-bonding interactions of small terminase with bases of DNA are formed solely by the DNA-binding substructure. The two helices forming this substructure position the sidechains of Arg128, which lies at the N-terminal end of the C-terminal helix, and Arg7 which is located in the NTA adjacent to the N-terminal helix, so they can reach into and contact bases in the major and minor grooves respectively. Arg128 forms bidentate hydrogen bonds with the base of G at position 21 in the center of the small terminase binding site. (Fig. 3*C*). Arg7 forms bifurcated hydrogen bonds with T16 at the 5′ end of the small terminase binding site (in the top strand; Fig. 3*C*). The position of both arginine sidechains is stabilized by ionic interactions with negatively charged residues Glu126 and Asp9 for Arg128 and Arg7, respectively. A similar mode of protein–DNA interaction was observed for the integration host factor interacting with the minor groove of DNA (29). In both pairs of positively/negatively charged residues a serine residue (Ser127 and Ser8) lies between the residues, and its sidechain makes a hydrogen bond to the phosphate backbone of DNA (Fig. 3*C*). Additionally, in the NTA the sidechain of Ser10 makes a hydrogen bond to the other side of the major groove to the Ser8 contact, thus stabilizing the interaction of the NTA with DNA that positions Arg7 in the minor groove. The DNA is bent to an angle of 34° (Fig. 2*F*) at the point at which Arg128 forms hydrogen bonds with G at position 21 (Fig. 3*C*). The bending results in distortion of the DNA away from B-form with the minor groove widened and the major groove narrowed at the region centered on position 21 (*SI Appendix*, Fig. S11). This deformation is further stabilized by the interaction of Lys132 with the phosphate backbone on the opposite side of the major groove to Ser127. There are two more residues with positively charged sidechains in the NTA—Arg5 and Lys4. While the density for the backbone in this region clearly shows that the NTA extends along the minor groove, there is no clear density to unambiguously define where these side chains contact DNA, suggesting the interaction could involve either contact to bases in the minor groove or the phosphate backbone.

### Influence of DNA Sequence on Binding.

**Fig. 4.**
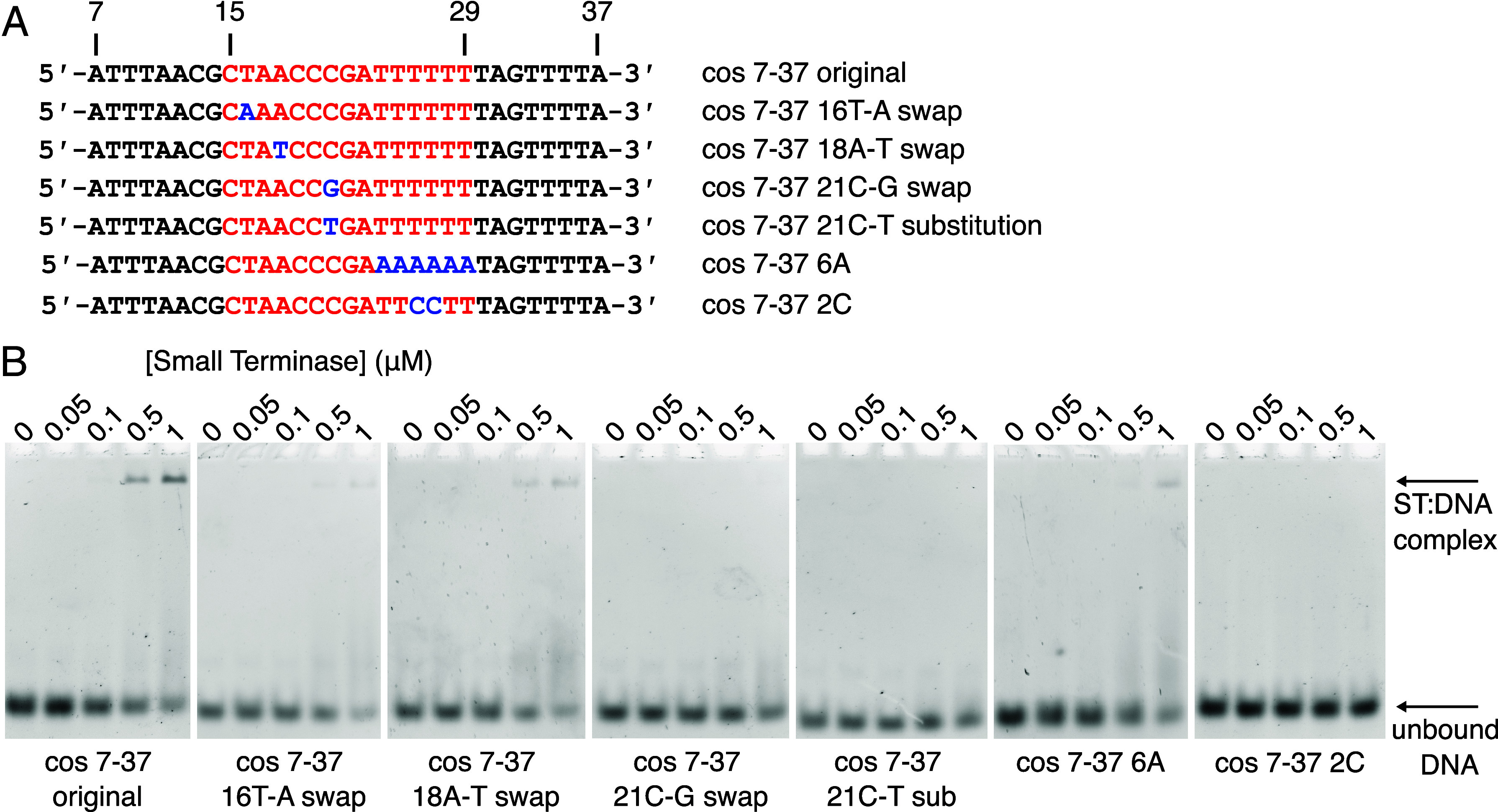
Interaction of small terminase with DNA mutants. (*A*) DNA sequences showing mutations in the small terminase binding site. The small terminase binding site is shown in red. Mutations are highlighted in blue. (*B*) EMSA of wild type and mutant DNA oligonucleotides with small terminase (ST).

We investigated the importance of the nucleotide sequence of the binding site in the minor and major grooves (Fig. 4). Swapping the orientation of the C:G base pair in position 21 or substitution of the C:G base pair for a T:A base pair reduced DNA binding to a level undetectable by EMSA. In contrast, swapping the orientation of the A:T base pairs in position 16 (where Arg7 contacts DNA) or position 18 (a possible interaction with Lys4) results in a smaller reduction in DNA binding—as expected because of the similarity between A:T and T:A moieties exposed in the minor groove (30). This confirms that Arg128 in the C-terminal helix of the DNA-binding substructure plays a crucial role in specific protein–DNA interaction.

To further investigate the influence of the DNA sequence, and any intrinsic deformability, on the specificity of small terminase binding, we designed two mutant DNA oligonucleotides, modifying the composition of the region of the binding site located in the central channel (Fig. 4). As would be expected given the non-sequence-specific interaction between small terminase and DNA within this region, swapping the orientation of A:T base pairs at positions 24 to 29 had little influence on DNA binding. In contrast, introducing two G:C base pairs at positions 26 to 27 in the middle of the A-tract abolished DNA binding. This indicates that while there are no base-specific contacts within the tunnel, the flexibility of the DNA across the small terminase binding site, including the flanking regions, accorded by this A:T rich region (31), is essential for binding.

### Role of Positively Charged Residues in Interaction with DNA.

To define the contribution of arginine and lysine residues in small terminase:DNA interaction we produced mutant proteins replacing several of these residues by alanine. Three residues within the central channel were mutated: Lys100, Arg107, and Arg114. Additionally, Lys4, Arg5, and Arg7 in the NTA of the DNA-binding substructure were individually mutated and a triple mutation of all three residues was also produced. Finally, Arg128 in the DNA-binding substructure was mutated. Size exclusion chromatography (*SI Appendix*, Fig. S12) and circular dichroism (CD) (*SI Appendix*, Fig. S13) confirmed mutant proteins were correctly folded and assembled into nonamers.

**Fig. 5.**
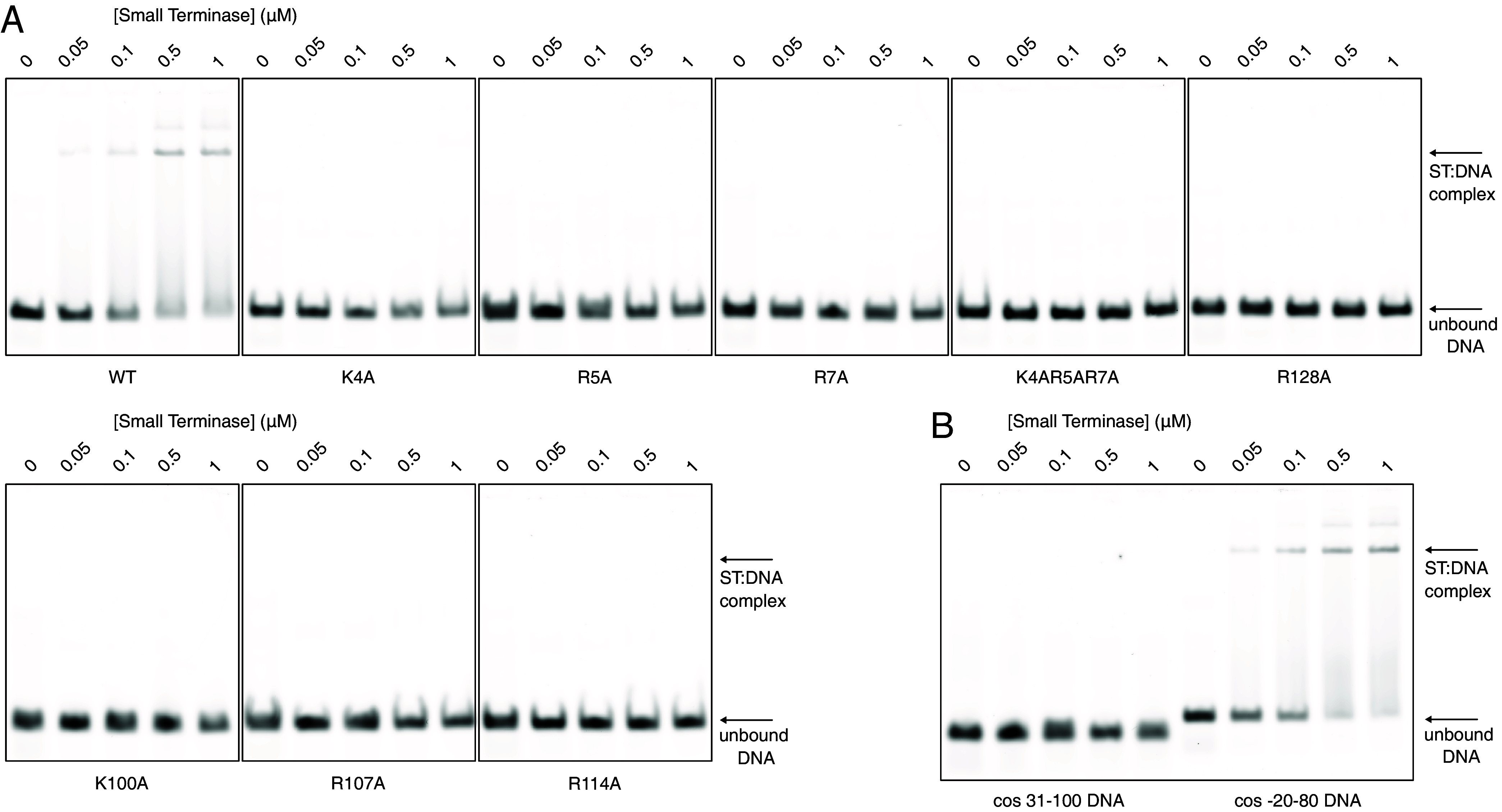
Interaction of small terminase mutants with DNA. (*A*) EMSA of WT small terminase (ST) and mutants with 100 bp DNA containing the small terminase binding site labeled with Alexa 647 (cos −20-80). (*B*) EMSA of small terminase with 100 bp (cos −20-80) DNA and 70 bp DNA lacking the small terminase binding site labeled with Cy5 (cos 31-100).

No DNA binding by any of the small terminase mutants could be detected by EMSA using a fluorescently labeled 100 bp DNA duplex containing the small terminase binding site (Fig. 5*A*). We attempted to measure the equilibrium binding constant for wild type (WT) small terminase and selected mutants using microscale thermophoresis (MST) experiments with the same DNA (*SI Appendix*, *Supporting text* and Figs. S14 and S15). This is complicated by the mixture of non-specific and specific DNA binding recorded by MST, however, we can determine a rough estimate for the upper limit of the apparent equilibrium dissociation constant (K_app_) of at least 10^−7^ M (*SI Appendix, Supporting text*). To confirm the specificity of DNA interaction, a 70 bp DNA fragment missing the specific binding site, labeled with Cy5, was used in a control experiment (Fig. 5*B*). No binding to this DNA could be observed by EMSA, however a rough estimate of the K_app_ for nonspecific binding by WT small terminase, determined by MST, was in the range of 10^−5^ to 10^−4^ M, around two orders of magnitude weaker than for the 100 bp DNA containing the sequence-specific binding site (*SI Appendix, Supporting text*). Comparison of the raw MST data also revealed that the triple mutation of the positive residues Lys4, Arg5, and Arg7 in the DNA binding substructure, appeared to reduce binding affinity, as observed in EMSA (Fig. 5*A*), but likely retained nonspecific binding activity (*SI Appendix*, Fig. S14*C*). Conversely, mutation of Arg107, within the central channel of the small terminase nonamer most obviously reduced binding features characteristic of non-sequence-specific binding (*SI Appendix*, Fig. S14*D*).

## Discussion

### Comparison of Small Terminase Structures of Double-Stranded Bacteriophages.

We report an experimentally derived structure of a small terminase protein bound to DNA, unveiling structural events that occur during recognition of viral genomic DNA by the small terminase. To date, no common mechanism by which these proteins bind to DNA has been elucidated but several conflicting models have been proposed (11, 17, 19, 21). Our structure reveals that regions disordered in the absence of DNA are critical for DNA binding and illustrates how mechanisms proposed based on structures of DNA-unbound small terminase may miss vital details. The very N terminus is well defined only in the structures of small terminase from G20c and λ phage. Intriguingly there is at least one positively charged residue at the N terminus of all small terminases (*SI Appendix*, Table S3) suggesting that, as we observe for the HK97 small terminase, regions at the N terminus of small terminase from other bacteriophages, not visualized in the structures determined in the absence of DNA, may also become ordered upon DNA binding. This is supported by data for the Sf6 small terminase, where it was shown that a Lys6Ala mutant in the unstructured N-terminal region abolished DNA binding (21). In all small terminase structures determined to date, residues at the C terminus are disordered, but their number ranges from very few (such as in SF6) to more than 40 disordered residues (HK97, G20c, 44RR, E217). These unstructured regions are clearly visible as diffuse density in cryoEM 2D classes of E217 (14) and P74-26 small terminases (13). The only previous evidence that the C terminus was involved in DNA binding was for the P22 bacteriophage (18), but given the contribution to DNA binding from the C-terminal region of the HK97 small terminase where Arg128 makes a key sequence-specific contact with DNA, it seems possible that the unstructured C-terminal regions of the small terminase from other bacteriophages may also become ordered upon DNA binding.

In all small terminase proteins, the surface of the internal channel is positively charged (*SI Appendix*, Fig. S1), prompting speculation that, as for HK97, DNA may bind in the central channel. However, it is unlikely that this is the only mode of small terminase:DNA interaction in all bacteriophages. Certainly, for small terminases from bacteriophages T4 and SF6, the assembled central oligomerization domain showed significantly reduced DNA binding activity (11, 32). In the case of T4, these experiments used EMSA and we note that this technique was also unable to detect binding by HK97 small terminase when mutations were made in the DNA-binding substructure (Fig. 5*A*). However, T4 small terminase tunnel mutants did exhibit minimal effect on DNA binding (32). Crucially for SF6, there is experimental evidence indicating that DNA does not interact with residues in the central channel (11). It is also of note that diameters of the central channels vary between the small terminases from different bacteriophages (*SI Appendix*, Fig. S16) and while the channel of small terminase from G20c, P74-26, E217, 44RR, and HK97 is wide enough to accommodate B-form DNA without any clashes, in small terminases from P22, SF6, SPP1, Sf6, PaP3, and pHBC6A51, the channel appears to be too narrow for DNA to pass through.

### HK97 Small Terminase Recognizes DNA Using a Unique “Arginine Clamp”.

To our knowledge, the structure of HK97 small terminase bound to DNA reveals a hitherto unknown mode of DNA binding. Our data suggest that both the bending of DNA and the interaction with Arg128 are crucial for sequence-specific DNA recognition by HK97 small terminase because mutation of either Arg128 or the guanidine at position 21 this residue forms a hydrogen bond to abolishes DNA binding, while swapping DNA bases in the minor groove had a minor influence on interaction (Fig. 4). The positioning of two arginine residues into the major and minor grooves of the DNA form an arginine clamp that locks the bent conformation of DNA into place (Fig. 3). Further residues from the DNA-binding substructure hydrogen bond to the phosphate backbone and stabilize the clamp’s grip on DNA. This interaction relies on the inherent deformability of the overall DNA sequence, facilitated by the run of A:T base pairs (31, 33) within the central channel adjacent to the bend, since disrupting the propensity for bending of this sequence by the inclusion of C:G base pairs abrogated small terminase binding (Fig. 4). The clamp ensures that small terminase tightly binds to its binding site.

### A Model for Packaging Termination in HK97 Bacteriophage.

**Fig. 6.**
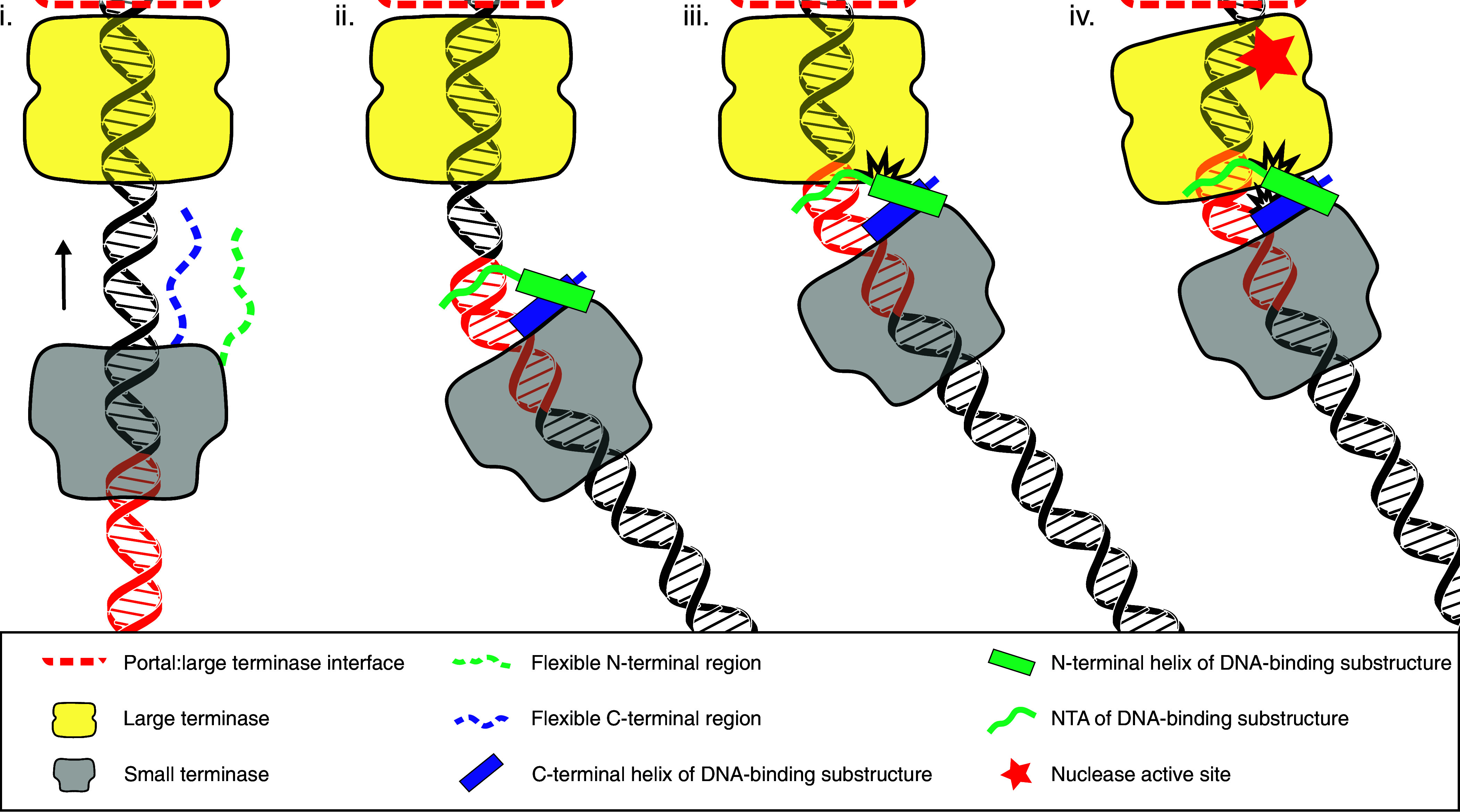
Proposed mechanism of DNA recognition and packaging termination. Roman numerals refer to stages described in the text. The small terminase binding site is highlighted in orange-red on the DNA. Arrows indicate the direction of the DNA movement. For clarity, only one copy of the flexible N- and C-terminal regions of the nine protomers in small terminase is shown.

The small terminase is essential for termination of packaging at the *cos* sequence (3, 6), and data presented here enable us to suggest a model of packaging termination in *cos* bacteriophages, involving four intermediate structural events, (i) to (iv) in Fig. 6. We propose that during DNA packaging the small terminase encircles the DNA through non-sequence-specific interactions mediated by the positively charged residues lining the surface of the nonamer. However, contrary to recent proposals for λ (34), we do not envisage that HK97 small terminase functions as a “sliding clamp” remaining an inherent part of the terminase complex throughout the packaging process. Indeed, while the large terminase mechanically translocates DNA into the capsid [event (i)], the symmetry mismatch with the pentameric large terminase (6) and the dynamic nature of conformational changes within large terminase during DNA translocation (27, 35) result in the large terminase pushing the small terminase along DNA. During this process, the transient nature of the interface between the large and small terminase proteins, mediated in part by their respective non-sequence-specific interactions with the DNA, would allow the small terminase to rotate with respect to the large terminase and/or DNA. Such a dynamic interface is consistent with the inability to assemble terminase complexes from most bacteriophages in vitro in the absence of procapsids and/or DNA with the notable exception of λ where the terminase can be purified as a heterooligomer (36). We postulate that during this process the flexible N- and C-terminal regions of small terminase act as molecular sensors surveilling the DNA as it exits from the central channel. As the 9 bp long A-tract begins to exit the channel, the flexibility of the junction between this and the adjoining C:G base pairs, allows the DNA to bend [event (ii)]. Shape matching of the bent DNA with the C-terminal region of one protomer positions its α-helix so that the side chain of Arg128 can insert into the major groove, specifically interacting with the base of G at position 21. Concurrently, the N-terminal region of the adjacent protomer then folds into an α-helix that stacks alongside the α-helix from the C terminus to complete the DNA-binding substructure with the NTA inserting into the minor groove and Arg7 making contact with T16. This closes the arginine clamp which locks small terminase onto DNA at this position. During event (iii), the bent conformation of the bound DNA results in an interaction between small and large terminase in which the central axes of the circular assemblies of the small and large terminase oligomers no longer align, stalling DNA translocation. As the bent region of DNA is pulled further into the central channel of the large terminase [event (iv) Fig. 6], it becomes loaded into the active site of one of the five nuclease domains triggering the switch of large terminase from DNA translocation to nuclease activity. This mechanism, based on structural and biochemical data reported here, is also supported by the previously observed stalling of translocation in HK97 in vitro at experimentally induced low packaging velocities that occurs only when both small terminase and a *cos* site is present (6, 27). Pausing at the *cos* site can only arise from interaction of the small terminase bound at its specific recognition site with large terminase. The specific recognition of G at position 21 by Arg128 and the adjacent A-tract via the shape of the DNA ensure that stalling and subsequent triggering of nuclease activity occur only when the *cos* cleavage site is accommodated at the nuclease active site of the large terminase. Cleavage at the *cos* site was shown to be stimulated by the HNH endonuclease gp74 in vitro (6, 37). The 10 nucleotide 3′-overhang at the *cos* cleavage site could result from cleavage of one strand by the large terminase and the other by gp74. Alternatively, after the first strand is cut by the nuclease domain of large terminase subsequent DNA translocation as the arginine clamp is released from the binding site before the second strand is cut produces the 3′-overhang. Finally, the terminase complex dissociates from the capsid vertex ready for the next packaging event.

In contrast to *cos* phages, in *pac* phages the small terminase is not required for termination of packaging at a specific site (8). However, the increased efficiency of small terminase-directed termination at low packaging speeds in *cos* phages suggests that headful pressure signaled via the portal protein, reported for *pac* phages, may also play a role in *cos* phages (38, 39).

## Conclusions

In this work, we defined the small terminase binding site in the HK97 genome which enabled us to determine the structure of a viral small terminase protein in complex with DNA. The structure reveals a hitherto unknown mode of DNA recognition by a circular protein that can slide along DNA. Upon encountering the specific DNA binding sequence, two disordered regions at the N terminus and C terminus, respectively, of two adjacent protomers, fold into α-helices and form a DNA-binding substructure that locks small terminase onto DNA. The unique properties of this protein allowing threading along DNA while transiently surveilling its sequence until a specific site is reached and arrested by the protein, are likely to make it a valuable tool for biotechnological applications. The structural findings, unveiling molecular events that occur during viral DNA recognition by the *cos* bacteriophage HK97, offer insights into understanding mechanisms of other systems featuring circular DNA-binding proteins.

## Materials and Methods

### Cloning.

The gene encoding HK97 small terminase (residues 1 to 161) was cloned in Champion pET SUMO (small ubiquitin-like modifier) vector (ThermoFisher) (28). Mutants of small terminase were produced by site-directed mutagenesis with the original plasmid as a template and a pair of oligonucleotides (Eurofins Genomics) containing mutated region.

### Protein Expression and Purification.

Small terminase was overexpressed in *E*. *coli* BL21(DE3) Gold pLysSRARE cells. Cells were grown in 1L LB media supplemented with 30 µg/mL kanamycin and 34 µg/mL chloramphenicol at 37 °C till OD_600_ reached 0.6. Then the flasks were cooled on ice and expression was induced with 0.5 mM IPTG. Cells were incubated at 16 °C overnight and harvested by centrifugation. The cell pellet was frozen at −80 °C. Cells were resuspended and lysed by sonication in buffer A: 50 mM Tris-HCl, pH 7.5, 1 M NaCl, 5% (v/v) glycerol, 30 mM imidazole supplemented with 100 µM 4-(2-aminoethyl) benzenesulfonyl fluoride, 0.5 µM leupeptin, 0.7 µM pepstatin, 100 µg/mL lysozyme, 10 µg/mL RNaseA, 2.5 µg/mL DNase, 1 mM MgCl_2_. After centrifugation for 1 h at 18 K rpm, cell lysate was applied to HisTrap FF Ni column (Cytiva). To reduce nucleic acid contamination, the column was washed with 10 column volumes (CV) of buffer A (resuspension buffer without protease inhibitors and other additives), followed by 5 CV of high salt buffer (25 mM Tris-HCl, pH 7.5, 3 M NaCl). After re-equilibration of the column with buffer A, the protein was eluted with 50 mM Tris-HCl, pH 7.5, 1 M NaCl, 5% (v/v) glycerol, 0.5 M imidazole. Eluted protein was diluted twice with 25 mM Tris-HCl, pH 7.5 to reduce NaCl concentration to 0.5 M and incubated with 1:100 (w/w) SUMO protease overnight at 4 °C with addition of 2 mM DTT. Cleaved protein was diluted further to 0.2 M NaCl concentration and applied to cation exchange 10/10 Mono S column (GE Healthcare). Small terminase was eluted in two peaks with 0.2 to 1 M NaCl gradient. Fractions from each peak were concentrated separately in Vivaspin concentrators (Sartorius) with MWCO 30 K cutoff and loaded onto a Superdex 200 10/300 column (GE Healthcare) in 25 mM Tris-HCl, pH 7.5, 1 M NaCl buffer. Finally, a heparin column (GE Healthcare) was used for the polishing step. Protein was loaded on the column in 25 mM Tris-HCl, pH 7.5, 0.15 M NaCl buffer and was eluted with a linear gradient 0.15 to 1 M NaCl. Protein was concentrated and flash frozen in liquid nitrogen and stored at −80 °C.

Small terminase mutants were expressed in the same way as WT protein with the following modifications. For Ni affinity purification a batch binding method was used. Cleared cell lysates were mixed with 5 mL Ni-NTA affinity resin (Generon) equilibrated with buffer A and incubated by rotating the tubes for 30 min at room temperature (RT). Resin was poured into 10 mL empty PD-10 columns (GE Healthcare) and all washes and elution were performed by gravity flow with buffers as used for WT purification. Additionally, a heparin column was used after SUMO protease cleavage instead of a cation exchange column. This step helped to separate cleaved protein from SUMO tag as well as minimize nucleic acid contamination. Finally, all mutants were further purified by size exclusion chromatography on a Superdex S200 10/30 column in 25 mM Tris-HCl, pH 7.5, 1 M NaCl, concentrated and frozen in liquid nitrogen.

### Preparation of DNA Oligonucleotides.

Fluorescent DNA fragments of 100 bp (cos −20-80) and 70 bp (cos 31-100) were produced by PCR with DreamTaq DNA polymerase (ThermoFisher Scientific). The forward primer (Eurofin Genomics) was covalently linked with Alexa 647 for 100 bp DNA and the reverse primer with Cy5 for 70 bp DNA. PCR settings: initial cycle 94 °C (30 s) followed by 35 cycles of 94 °C (10 s), 60 °C (30 s) and final extension of 72 °C (5 min). A plasmid containing 784 bp segment around the *cos* site (−312 to +472) was used as template in all reactions at a final concentration of 2 pg/µL (28). The final PCR products were purified using a PCR clean-up kit (Macherey-Nagel).

Short oligonucleotides for EMSA and complex formation (Sigma-Aldrich) were reconstituted with TE buffer (10 mM Tris-HCl, pH 8.0, 1 mM EDTA) to a final concentration of 200 µM. Equimolar quantities of complementary oligonucleotides were mixed in a PCR tube and annealed in a heating block (5 min at 95 °C followed by slow cooling over 2 h to RT). The quality of annealing was checked on 10% acrylamide native gel. Annealed oligonucleotides were diluted with water to 10 µM before use.

### EMSA.

To identify the minimal binding site for each native gel a master mix (MM) was prepared containing small terminase in 25 mM Tris-HCl, pH 7.5, 200 mM NaCl, 10 mM MgCl_2_ buffer. 8 µL of MM were aliquoted into each tube and then 2 µL of 10 µM DNA was added followed by thorough mixing. Final concentrations of small terminase and DNA in the 10 µL reaction were 1 µM and 2 µM respectively. Tubes were incubated at RT for 3 h. 3 µL of loading buffer (50% glycerol, Orange G) was added to each tube, mixed, and spun for 1 min at 13 K rpm before loading on 6% native gel. A gel with acrylamide:bis-acrylamide 99:1 ratio was prepared using running buffer 25 mM Tris-HCl pH 8.3, 192 mM glycine, 200 mM Na_2_SO_4_, and 10 mM MgSO_4_ and poured into empty 1.5 mm thick gel cassettes (ThermoFisher Scientific), which made handling of low percentage gels easier. Gels were run overnight at 4 °C with current of 15 mA to prevent overheating. Gels were rinsed with TAE buffer followed by staining with 0.2% ethidium bromide in TAE buffer for 30 min and imaged with GelDocXR+ (BioRad). This procedure was modified for characterization of the effect of mutations within the binding site as follows: 8 µL of MM containing DNA were aliquoted into five tubes and then 2 µL of serial dilutions of small terminase were added, resulting in final protein concentration range of 0.05 to 1 µM and DNA concentration of 0.5 µM. Samples were incubated for 2 h at RT and run on 6% native gel at 4 °C for 4 h with current of 60 mA.

Thirty percent acrylamide:bis-acrylamide (99:1 ratio) solution was prepared by mixing 7.4 mL of 40% acrylamide (BioRad), 1.5 mL of 2% bis-acrylamide (Biorad) and 1.1 mL of water. Such acrylamide:bis-acrylamade solution was used to prepare gels with a larger pore size to facilitate movement of the large protein:DNA complex through the gel.

EMSA for small terminase mutants was performed as described above. 100 bp DNA labeled with Alexa647 (cos −20-80), was used at a final concentration of 20 nM, 70 bp DNA lacking the small terminase binding site labeled with Cy5 (cos 31-100) was used at a final concentration of 40 nM. 8 µL of MM containing fluorescent oligonucleotides in 25 mM Tris, pH 7.5, 200 mM NaCl, 10 mM MgCl_2_ was aliquoted into tubes, then 2 µL of serial dilutions of small terminase were added, resulting in final protein concentration range of 0.05 to 1 µM. Samples were incubated for 2 h at RT and run on 6% native gel at 4 °C for 4 h with current of 60 mA and scanned with GE Amersham Typhoon-5 fluorescence gel and blot scanner (GE Healthcare) with red laser.

### Far-UV CD Spectropolarimetry.

Proteins (WT and mutants) were diluted to a final concentration of 0.3 mg/mL with 25 mM Tris-HCl, pH 7.5, 100 mM Na_2_SO_4_ in a total volume of 400 µL. Spectra were collected on a Jasco J-1500 CD spectrometer from 260 nm to 195 nm with 1 mm path length.

### MST.

Protein and DNA were diluted in reaction buffer: 25 mM Tris-HCl, pH 7.5, 200 mM NaCl, 10 mM MgCl_2_, supplemented with 0.005 to 0.01% Tween 20 (NanoTemper Technologies). To minimize pipetting errors 1:1 serial dilutions were performed such that protein was diluted in 12 nM DNA solution. For each MST run, 2× stocks of small terminase and fluorescent DNA oligonucleotides were prepared at 16 µM and 24 nM, respectively. Stocks were spun for 3 min. DNA was diluted twice with reaction buffer to 12 nM and then 10 µL was aliquoted in 15 tubes. 10 µL of each 2× stock was mixed in a separate PCR tube for the highest protein concentration followed by serial dilution in 15 consecutive tubes. Tubes were spun again for 5 min at 13 K rpm before loading in Premium Monolith NT.115 capillaries (NanoTemper Technologies). This produced a series of 16 samples with small terminase concentration ranging from 0.24 nM to 8 µM and constant DNA concentration of 12 nM. For one run a 2× stock of WT small terminase at 32 µM was used resulting in final protein concentrations ranging from 0.48 nM to 16 µM. MST was measured using a Monolith NT.115 instrument (NanoTemper Technologies) at an ambient temperature of 20 °C. Instrument parameters were adjusted to 60% LED power and medium MST power (40%). Data from three or more independently pipetted measurements were analyzed (MO.Affinity Analysis software version 2.3, NanoTemper Technologies) using the signal from an MST-on time of 5 s.

### Small Terminase:DNA Complex Formation and CryoEM Sample Preparation.

Small terminase:DNA complex was prepared at 1:2 protein:DNA ratio by mixing small terminase with 31 bp DNA (cos 7-37) in reaction buffer 25 mM Tris-HCl, pH 7.5, 200 mM NaCl, 10 mM MgCl_2_. After incubation for 3 h at RT, it was further diluted with reaction buffer to 400 µL and final concentration of small terminase and DNA 11.9 µM and 24 µM, respectively. The small terminase:DNA mixture was loaded onto a S200 10/30 column (GE Healthcare) equilibrated with reaction buffer and run at 0.5 mL/min. 100 µL fractions were collected. The most concentrated fraction was spun for 5 min at 13 K rpm before applying 3 µL to UltraAuFoil R1.2/1.3 gold support grids (Quantifoil). Prior to sample applications grids were glow-discharged for 3 min in Pelco easiGlow glow-discharger (Pelco) at 20 mA, 0.38 mBar. Grids were blotted at 4 °C and 100% relative humidity for 2 s with −20 blot force and vitrified by plunging into liquid ethane using the FEI Vitrobot Mark IV (Thermo Fisher Scientific). Grids were prepared within 1 h of completion of the gel filtration run. Complex formation was later confirmed by running fractions on native acrylamide gel.

### CryoEM Data Acquisition.

Initial screening of grids was performed at the University of Sheffield on a Tecnai Arctica (FEI) 200 kV electron cryomicroscope. Two separate datasets were collected from the same grid using a Titan Krios microscope (FEI) operated at 300 kV and equipped with a Gatan K2-Summit detector and energy filter (a slit width of 20 eV was used) at the Astbury Biostructure Laboratory, University of Leeds. Movies were collected automatically with EPU (Thermo Fisher Scientific) software on Gatan K2-Summit detector operated in counting mode with calibrated pixel size of 1.07 Å. For the 1st dataset 682 movies were collected with target defocus range of 1.3 to 3.1 μm. Each movie comprised 50 frames with a total fluence of 53 e^−^/Å^2^ over 8 s, corresponding to a flux of 7.58 e^−^/pixel/s. For the 2nd data collection 2,085 movies were collected over a target defocus range of 1.0 to 2.4 μm with a total fluence of 54 e^−^/Å^2^ over 8 s, corresponding to a flux of 7.65 e^−^/pixel/s. To address any preferential orientation problem 995 movies were collected at 10° stage tilt and 71 at 20° stage tilt.

### CryoEM Data Processing (*SI Appendix*, Figs. S5–S7 and Table S1).

Both datasets were processed in RELION 3.1.2 (40). Micrographs from the first dataset were motion corrected using RELION’s implementation (41) of the MotionCor2 algorithm (42). After estimation of contrast transfer function (CTF) parameters with CTFFIND-4.1 (43) micrographs were manually sorted to eliminate empty and icy ones. Micrographs collected at defocus >3.8 µm were also discarded, resulting in 550 micrographs. Particle picking was performed with Topaz software (44). Initially, 2,700 particles were picked manually from a subset of 140 micrographs (denoised using Topaz). Topaz was then trained using these particle coordinates. The trained model was used to pick 250,798 particles from the complete dataset. Particles were extracted in 168 pixel boxes and downsampled to 2.50 Å/pixel. 2D classification of selected particles showed predominately side views, although there were some classes with possibly top/bottom views (*SI Appendix*, Fig. S5). After 2D classification the best classes (250,549 particles) were selected for 3D refinement. A map generated from the crystal structure of small terminase (PDB: 6z6e) (6) low-pass filtered to 10 Å was used as a reference. A mask around the map from the crystal structure was used to focus the refinement on small terminase. The resulting map was used for partial signal subtraction in order to improve the signal for focused classification of the DNA region. We created a funnel mask in Chimera (45) that masked DNA in the tunnel of small terminase as well as DNA above it.

3D classification focused on the region within this mask with no angular sampling and high T (T = 50) produced classes with different DNA orientations. The most prominent class with 38,582 particles was selected. Final resolution of the map after reverting subtraction, reconstruction, refinement, and postprocessing was 5.0 Å. Re-extracting particles in 224 pixel boxes and downsampling to 1.25 Å/pixel, followed by CTF refinement and Bayesian particle polishing (41) improved the resolution to 3.5 Å.

Movies from the second dataset were motion corrected and CTF parameters estimated as described for the 1st dataset (*SI Appendix*, Fig. S6). All icy and empty micrographs were discarded and additionally, micrographs collected at >3 µm defocus or with estimated astigmatism >600 Å were removed, resulting in 1,874 micrographs. Particles were picked using the same Topaz model as used for 1st dataset. 683,470 particles were extracted from those micrographs in 168 pixel boxes and downsampled to 2.50 Å/pixel. A round of 2D classification was used to clean the dataset, resulting in 669,682 particles. 3D classification was carried out with seven classes using the final model from the 1st dataset initially low pass filtered to 25 Å as the reference. Particles from the best class were re-extracted in 224 pixel boxes and downsampled to 1.25 Å/pixel followed by 3D refinement and then CTF refinement. Bayesian polishing was performed followed by another round of refinement. A final resolution of 3.9 Å was achieved. A second round of Topaz training was performed with selected particle coordinates from the final set of particles in the 2nd dataset (*SI Appendix*, Fig. S6). First, a subset of 517 micrographs containing more than 100 particles per micrograph was selected. 2D classification was performed on 62,616 particles from these micrographs and 250 random particles from 25 classes showing a variety of views were selected to create the set of 6,250 particle coordinates used for Topaz training. From this point, the workflow for both datasets was identical (*SI Appendix*, Fig. S7). Particle picking was performed with the new Topaz model from 550 and 1,874 micrographs for the 1st and the 2nd datasets respectively. 306,866 (dataset 1) and 837,519 (dataset 2) particles were picked and extracted in 168 pixel boxes and downsampled to 2.50 Å/pixel. These particles were cleaned by 2D classification followed by 3D classification. Particles from the best classes were re-extracted in 224 pixel boxes and downsampled to 1.25 Å/pixel followed by 3D refinement and Bayesian polishing with shiny particles extracted from movie frames in 320 pixel boxes and downsampled to 1.19 Å/pixel (final box size 288 pixels). After 3D refinement of the shiny particles the resolution of the maps was 3.1 Å and 3.2 Å for the 1st and 2nd dataset respectively. While performing Bayesian polishing, we noticed that particle movement graphs for each dataset looked very different. During the second data collection, a number of movies were collected with the stage tilted at 10° or 20°. Eventually, we discarded all micrographscollected with the stage tilted from this dataset. 3D refinement of 334,433 particles from combining both datasets was followed by another round of CTF refinement and 3D refinement leading to an overall resolution of 2.9 Å. The final set of 334,433 particles was split into two subsets: 174,040 particles from micrographs collected at defocus <2 µm and 160,165 particles from micrographs collected at defocus >2 µm. The subset of particles from micrographs collected at defocus <2 µm refined to identical overall resolution as the whole dataset. To improve the density for the N-terminal helix that contacts DNA, we performed focused 3D classification on the particles from micrographs collected at defocus <2 µm, splitting particles in two classes with no angular sampling and high T (T = 32) with partial signal subtraction using a mask containing the region of two helices and a segment of bound DNA (*SI Appendix*, Fig. S7). 3D refinement of 76,626 particles from the best class resulted in slightly lower overall resolution of 3.0 Å, but better resolved density for the N-terminal helices.

### Model Building and Refinement (*SI Appendix*, Table S2).

Atomic model building in the final map was performed in Coot (46) using crystal structure of HK97 small terminase (pdb 6z6e) as the initial model. Each chain was fitted individually with jiggle fit option. Ideal poly-alanine α-helices were fitted into the extra density and then side chains were built. Two segments of ideal double-stranded B-form DNA were fitted into DNA density and then joined together. It was possible to unambiguously assign the DNA sequence due to the high quality of the density for the middle region of DNA. Overall, 28 out of 31 DNA base pairs were modeled. No density was observed for residues 1 to 23 and 124 to 161 in 7 of the 9 protein chains. Real-space refinement was carried out in ISOLDE (47) followed by reciprocal space refinement with REFMAC5/Servalcat (48) using DNA restraints generated with libg in CCP-EM (49) and cycles of manual rebuilding in Coot (46). Model validation was done in CCP-EM using Validation:model software. All figures were generated using ChimeraX (50) and Chimera (45). To calculate the electrostatic potential, the PDB format files were converted to PQR format with the PDB2PQR server using the PARSE force field and assigned protonation states at pH 7.5. The APBS server (51) was used and 0.2 M of ions were included in the calculation. Surfaces of small terminases in *SI Appendix*, Fig. S1 were colored by default electrostatic potential algorithm in ChimeraX. Hydrogen bonds were estimated with PISA server (52). The angle of the DNA bend in Fig. 2*F* was calculated between the vectors obtained by aligning 10 bp fragments of ideal B-form DNA with DNA from the structure at the bottom and the top termini.

## Supplementary Material

Appendix 01 (PDF)

Dataset S01 (XLSX)

## Data Availability

Atomic coordinates and cryoEM maps have been deposited with the Protein Data Bank and Electron Microscopy Data Bank under accession numbers 8POP (53) and EMD-17794 (54) (map after focused classification), EMD-17818 (55) (consensus map), respectively. The raw movies have been deposited in the Electron Microscopy Public Image Archive under accession code EMPIAR-11620 (56).
