# Supplementary material for: Structural basis for DNA recognition by a viral genome-packaging machine
Source: Proc Natl Acad Sci U S A. 2024 Aug 8;121(33):e2406138121. doi: 10.1073/pnas.2406138121 (PMC11331095; doi:10.1073/pnas.2406138121)
Supplement: Supplementary file 1 — Appendix 01 (PDF) [file pnas.2406138121.sapp.pdf]

PNAS

Supporting Information for

Structural basis for DNA recognition by a viral genome-packaging machine

Maria Chechik, Sandra J. Greive, Alfred A. Antson, Huw T. Jenkins

Alfred A. Antson
Email: fred.antson@york.ac.uk

Huw T. Jenkins
Email: huw.jenkins@york.ac.uk

This PDF file includes:

- Supporting text
- Figures S1 to S16
- Tables S1 to S4
- Legend for Dataset S1
- SI References

Other supporting materials for this manuscript include the following:

- Dataset S1

Supporting Text

Protein binding in a non-sequence specific manner to DNA in MST data.

Titration of cos 31-100 DNA, lacking the specific binding site, with wild type (WT) small terminase (**Fig. S14*A*)** clearly shows an increase in fluorescence intensity with increasing protein concentration, indicative of protein binding to the DNA and increasing the size of the bound DNA complex. Since this DNA lacks the identified specific binding site, the binding observed is non-sequence specific (cartoons in **Fig. S14*A***). This type of binding results in successive multiple protein molecules binding to a single DNA molecule as the concentration of protein increases (cartoons in **Fig. S14*A***), until the entire segment of DNA is covered in protein, known as maximal saturation. However, maximal saturation requires a very high concentration of protein, which was not reached under the conditions of these experiments. At maximal saturation, fluorescence intensity should eventually reach a plateau and no longer increase as more protein is added. Multiple non-sequence specific binding events are much more likely to occur if the target DNA is longer than the minimal size of DNA required for binding (*1-3*). Since the length of the DNA (70 bp for cos 31-100) is greater than the minimal DNA binding sequence defined by EMSA (15bp; **Fig. 1** and **Fig. S2**), it is a reasonable assumption that more than one small terminase protein is binding to each dsDNA molecule under these conditions. Since the maximally bound state (fluorescence intensity plateau) requires very high concentrations of protein, it was not reached during these experiments, despite use of up to 10 μM protein. This means that the non-specific binding site size (number of base pairs covered by non-specifically bound protein) is not defined experimentally. Thus, the equilibrium dissociation constant (K_d) for non-specific binding events cannot be determined accurately.

We note that no binding activity is observed in the EMSA for the cos 31-100 DNA (**Fig. 5*B***) that does not contain a sequence specific DNA binding site. This is because EMSA experiments titrated DNA (20 nM) with protein up to 1 μM (final ratio 1:50), while the more sensitive MST experiments use a lower concentration of DNA (12 nM) which is titrated with up to 8-10 μM small terminase protein (final ratio >1:650).

MST data showing protein interaction with DNA through both sequence specific and non-sequence specific binding events.

Titration of cos -20-80 DNA, containing the specific DNA binding sequence (shown in magenta in cartoon) with WT protein is shown in **Fig. S14*B***. The base level of fluorescence (at extremely low protein concentration) for cos -20-80 (~850 AU) is increased compared with cos 31-100 DNA (~790 AU) due to the additional 30 bp of the cos -20-80 DNA. As for cos 31-100, the fluorescence intensity increases with protein concentration reflecting changes in the size of the bound DNA complex with protein binding. This is likely to be not only due to single binding events of small terminase to the DNA at the specific site (cartoon in **Fig. S14*B***), but also because of additional non-sequence specific small terminase protein binding events to the DNA (cartoons in **Fig. S14*B***) since the fluorescence intensity does not reach plateau under these conditions. Indeed, this additional non-sequence specific binding, seen in MST, agrees with the observation of a second, slightly larger band for a small terminase:DNA complex that appears in the EMSA experiments at high concentrations of WT protein (**Fig. 5*A*** (first panel) and **Fig. 5*B***). Such additional non-sequence specific binding has been seen in EMSAs of lac repressor binding to DNA (*4*).

As for the case for non-sequence specific binding above, experimental limitations mean that the individual equilibrium dissociation constants (K_d) for sequence specific and non-specific binding events cannot be determined accurately.

Titration of cos -20-80 DNA with mutant small terminase proteins are shown in **Fig. S14*C*,*D***. Despite the challenge of not being able to quantitatively analyse the MST data, these experiments reveal some very interesting features of the interaction between the mutant small terminase

proteins and cos -20-80 DNA. Although the triple mutant (K4AR5AR7A, **Fig. S14*C***) appears to bind less efficiently to the cos -20-80 DNA compared to the WT protein, it appears to retain significant non-sequence specific binding by MST since the fluorescence intensity clearly increases with increasing protein concentration, exhibiting a similar profile to non-sequence specific binding of the WT protein to cos 31-100 DNA. Confirmation that this mutant has lost sequence-specific binding activity is provided by the EMSA experiments (**Fig. 5*A***). Conversely, the maximal fluorescence intensity in the experiment where cos -20-80 DNA was titrated with the R107A mutant small terminase (**Fig. S14*D***) is < 1/5 of that observed for the WT protein over the same range of protein concentration. This implies that this mutation significantly reduces binding activity of the small terminase. This appears to impact the non-sequence specific DNA binding activity, since the fluorescence intensity profile with increasing protein concentration no longer resembles that of WT protein to cos 31-100 DNA. Since the non-sequence specific electrostatic contacts contribute to the binding affinity for the sequence-specific site, very little binding is observed by MST, and none by EMSA (**Fig. 5*A***).

Estimates of apparent equilibrium dissociation constant (K_{app}) for specific and non-specific binding events.

Rough estimates for the apparent equilibrium dissociation constants (K_{app}) can be determined from fitting the MST data with the standard single site equilibrium binding quadratic equation assuming that a single protein binds to the DNA (**Fig. S14*E,F***). The fraction of DNA bound f(c), at a given protein concentration (c) for a constant total DNA concentration (c_T) in the assay is defined as:

$$f(c) = \frac{(c+c_T+K_d)\pm\sqrt{(c+c_T+K_d)^2-4cc_T}}{2c_T}$$

This model is adjusted to account for normalized fluorescence (Fnorm) of bound and unbound DNA as follows:

$$Fnorm = \left(1-f(c)\right)unbound + f(c)bound$$

Individual datapoints for each set of experiments were globally fitted to this model using the Levenberg-Marquardt Algorithm in pro Fit 7 (Quantumsoft), allowing all parameters to float globally except for the DNA concentration (c_T), which was held at 12 nM for all datasets. Data and fits were plotted as ΔFnorm.

For the non-sequence specific binding case, this approach leads to an overestimation of the K_{app} on the order 40 to 50-fold (*1*), given that: (i) the size of the DNA is much larger than the likely binding site size; (ii) multiple binding events take place; and (iii) non-sequence specific DNA binding sites overlap such that the effective concentration of non-specific DNA binding sites is much higher than that of the full-length DNA (12 nM). Thus, the K_{app} for non-specific binding is more likely to be in the range of 10^{-5}-10^{-4} M.

In the case where there is also sequence specific interaction (cos -20-80 DNA), the specific site is bound first with a higher affinity (EMSA data). However, the weaker non-sequence specific binding events contribute to the maximum binding plateau, thus this approach gives an underestimation of the K_d. Knowing this, we can define an upper limit for the apparent equilibrium dissociation constant (K_{app}) of at least 10^{-7} M, roughly 2 orders of magnitude tighter than for the non-specific case.

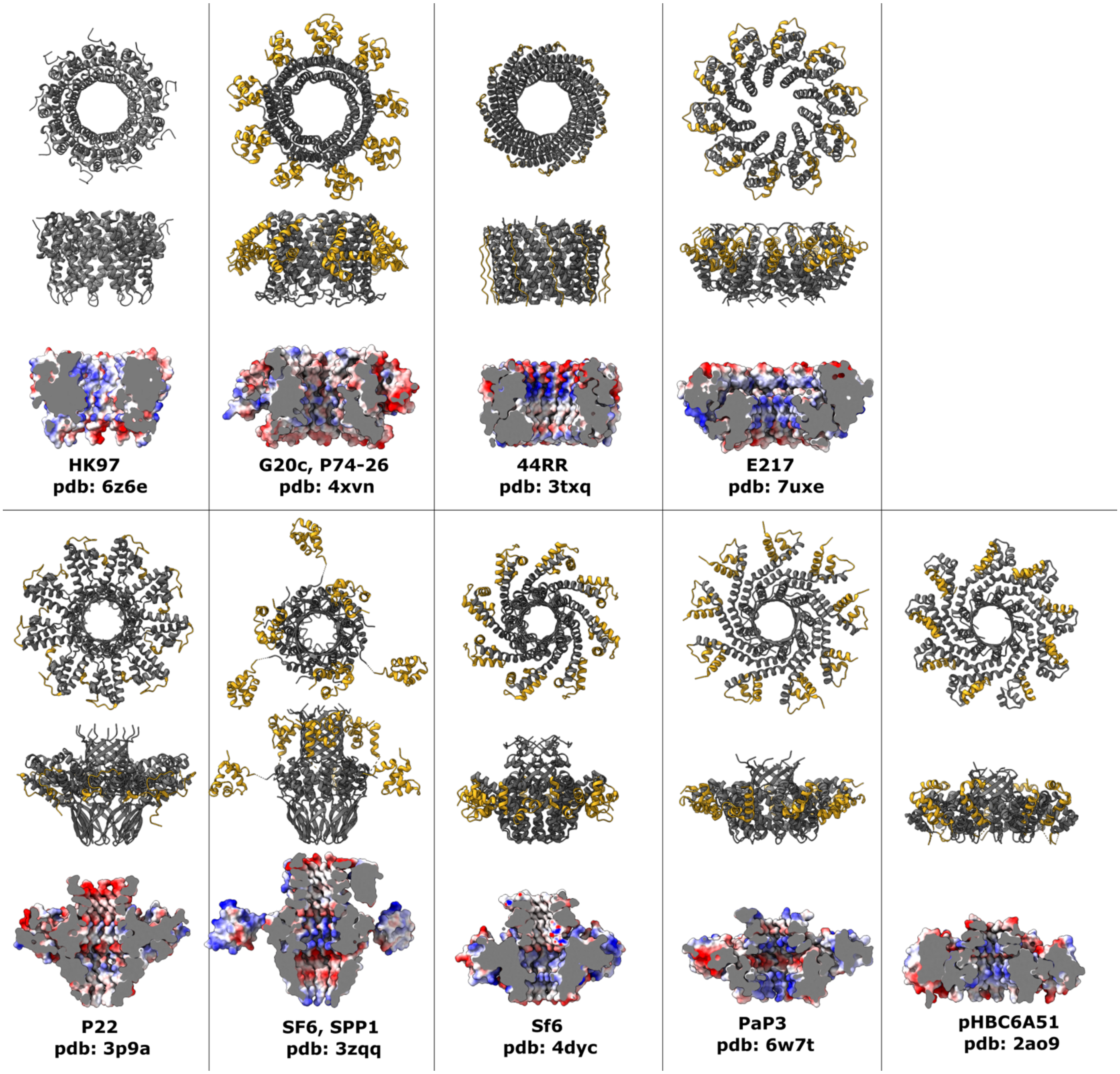

Fig. S1. Structures of small terminases deposited in PDB and EMDB. All structures are shown at the same scale: top and side view are presented with oligomerisation domain and C-terminus in grey and DNA-binding domain (confirmed experimentally or predicted) in gold. Below is the cross section of the same structure coloured by electrostatic potential (red - negatively charged, blue - positively charged, range -16.7 to +16.7 kT/e).

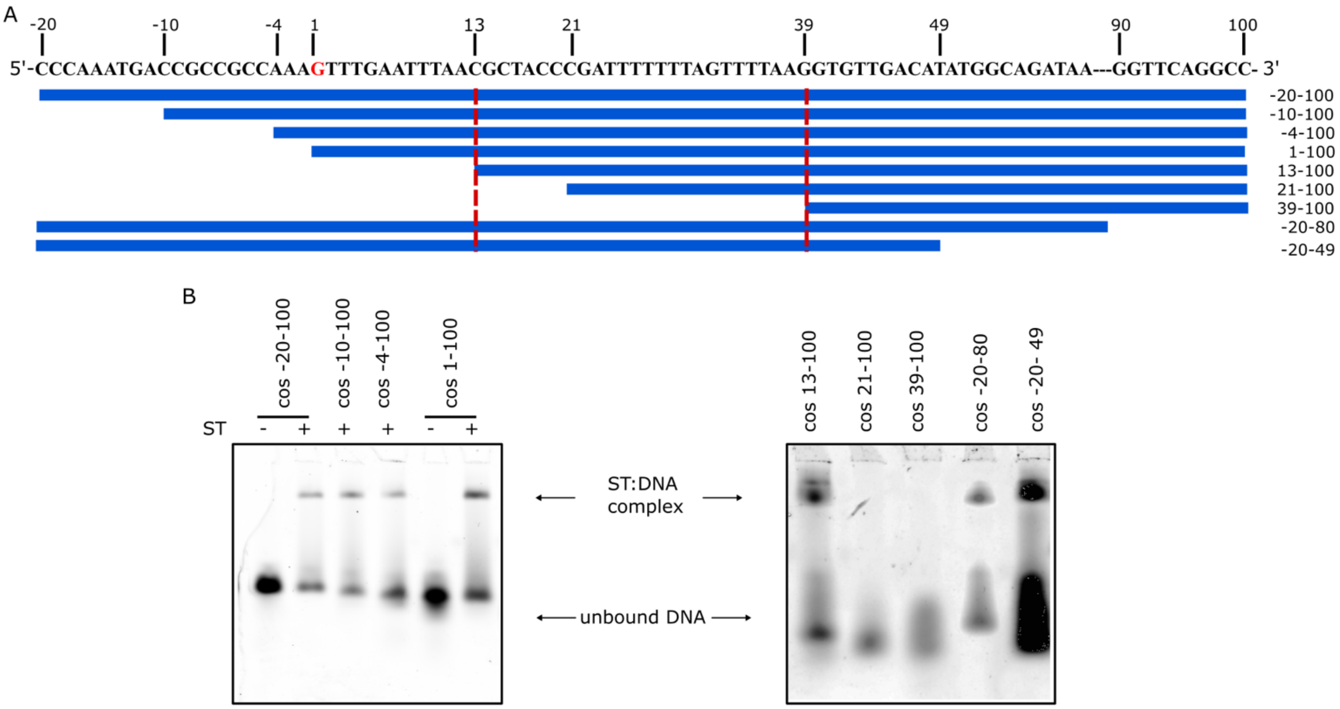

Fig. S2. Determination of small terminase binding site. (**A**) Sequence of HK97 putative small terminase (ST) binding site showing oligonucleotides used. Position 1 - cleavage site during genome packaging. The minimal small terminase binding site is marked with red dotted lines. (**B**) EMSA of interaction of fluorescent oligonucleotides spanning different regions of the putative binding site with small terminase.

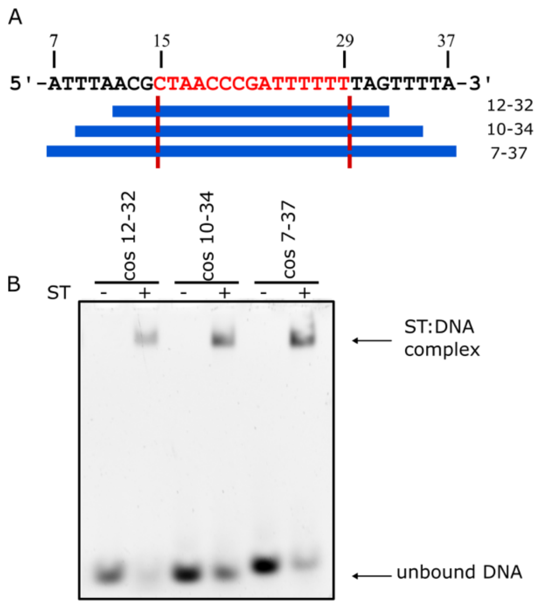

Fig. S3. Oligonucleotide optimisation for small terminase:DNA complex formation. (**A**) Sequence of small terminase (ST) binding site showing oligonucleotides used. (**B**) EMSA of the interaction of these oligonucleotides with small terminase.

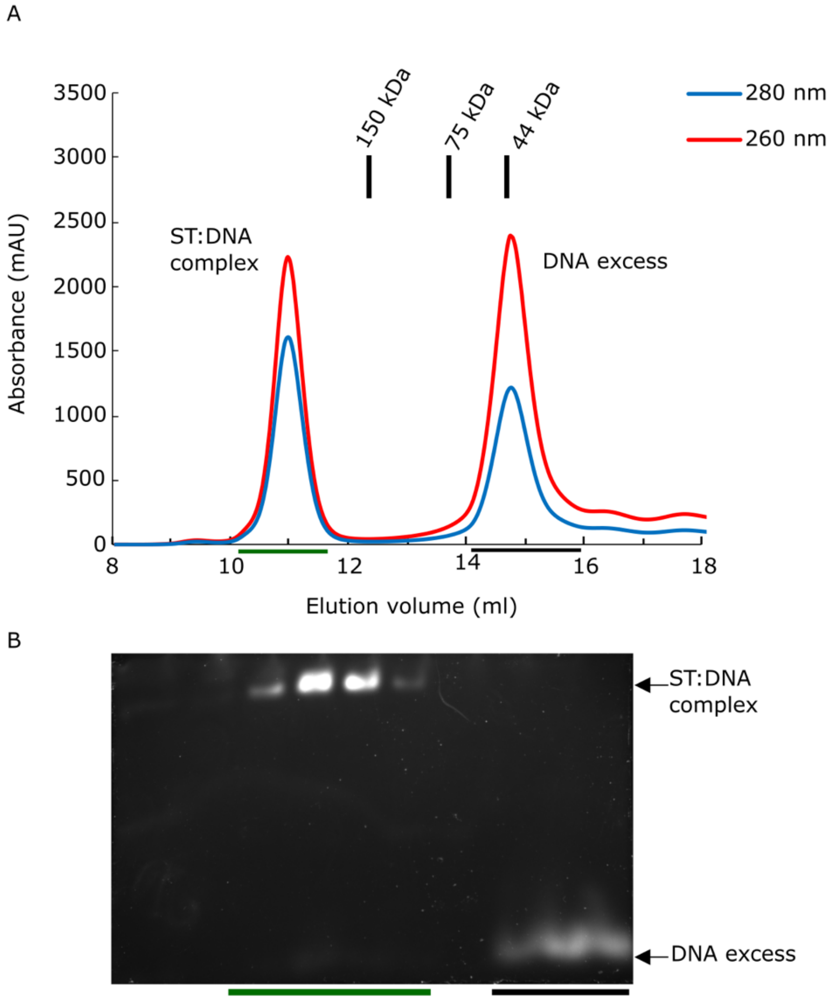

Fig. S4. Purification of small terminase:DNA complex on S200 10/30 column. (**A**) Analytical size exclusion chromatography profile. (**B**) Native PAGE, stained with ethidium bromide, with elution fractions for the complex (green bar) and excess of DNA (black bar) labelled.

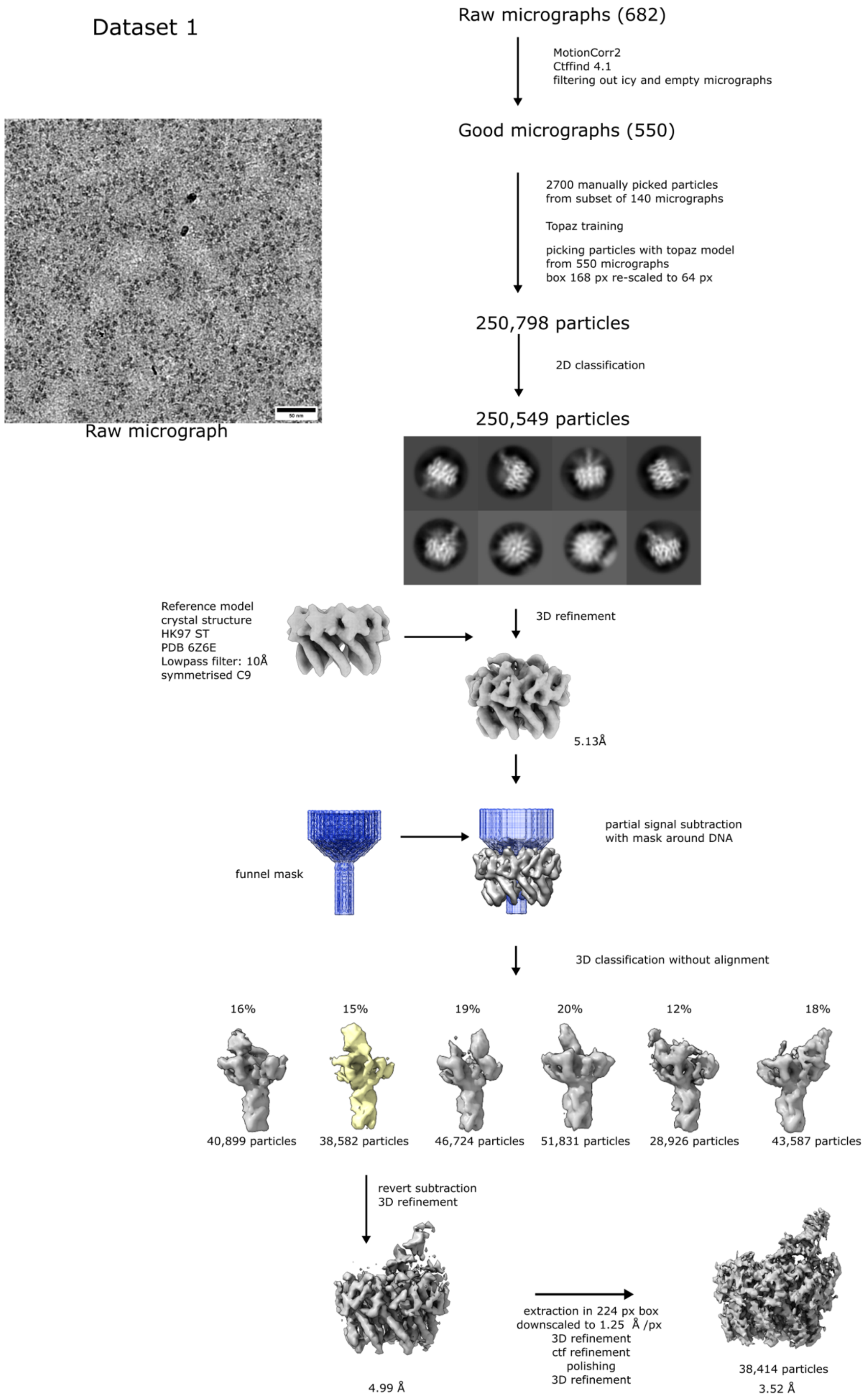

Fig. S5. CryoEM processing flow chart for dataset 1. Overview of initial cryoEM processing steps for small terminase:DNA complex dataset 1. Mask used in focused classification with partial signal subtraction is shown in blue. A representative micrograph showing particle distribution (scale bar is 50 nm) and a selection of 2D class averages representing different particle views is shown.

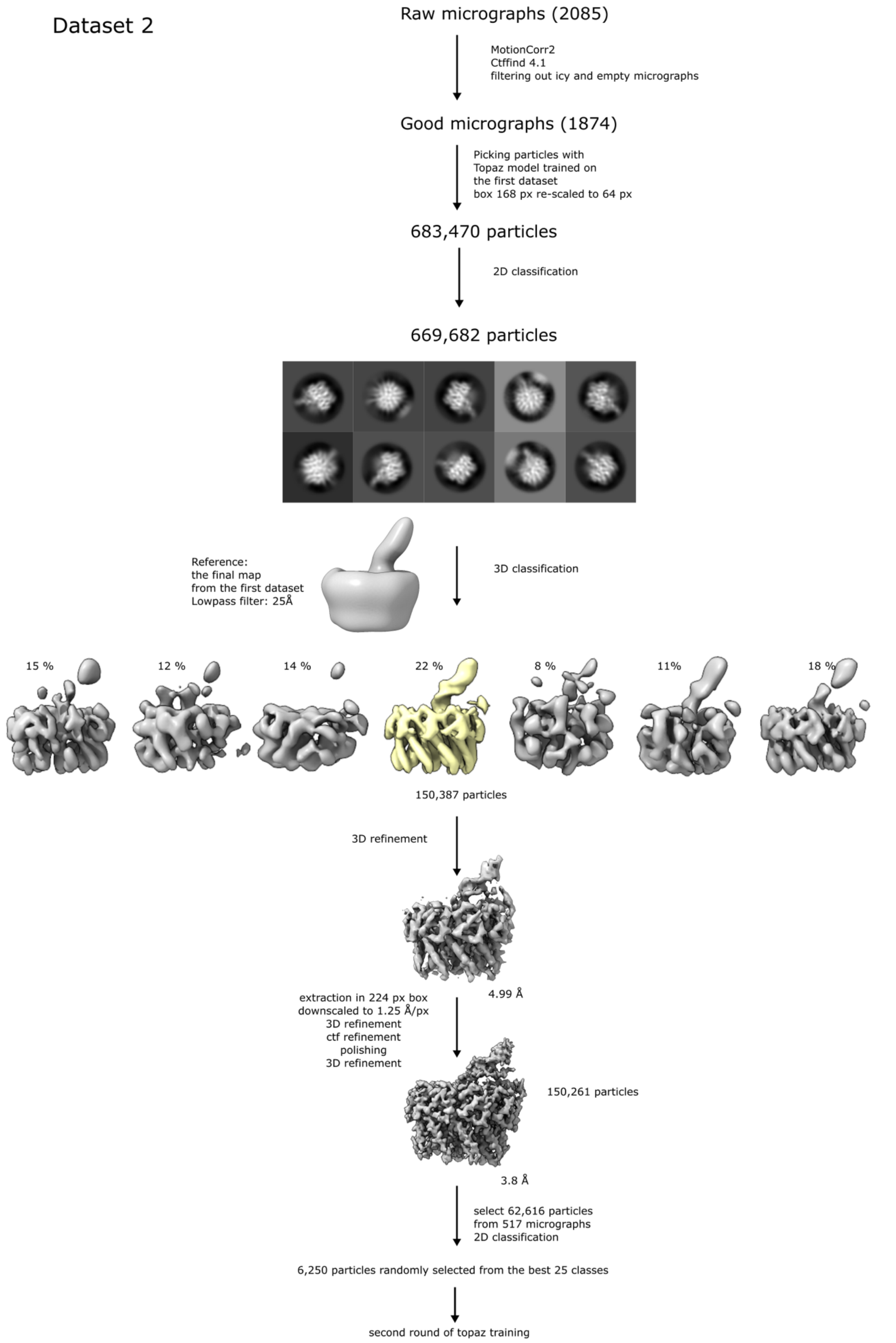

Fig. S6. CryoEM processing flow chart for dataset 2. Overview of the initial cryoEM processing steps for small terminase:DNA complex dataset 2. A selection of 2D class averages representing different particle views is shown.

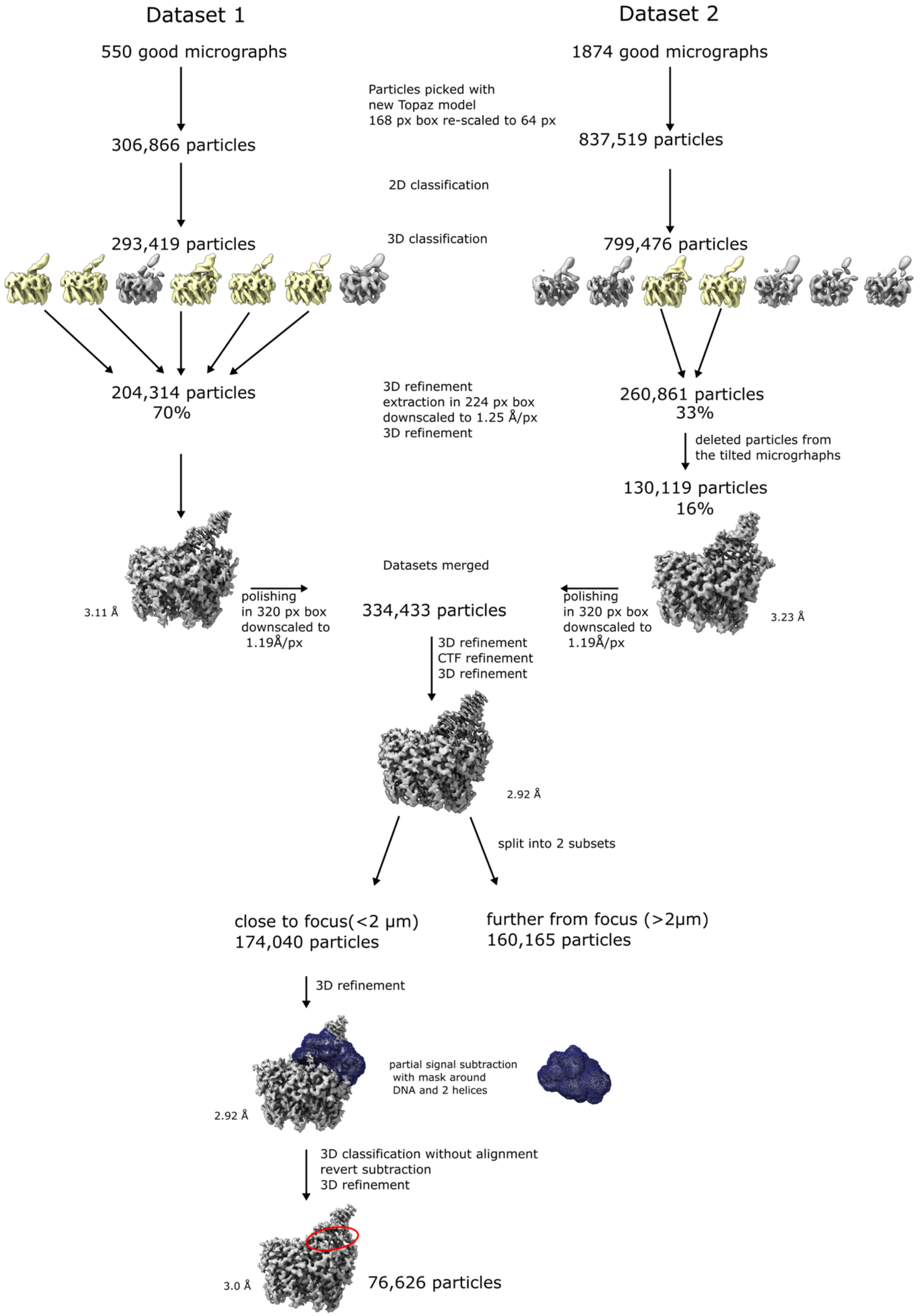

Fig. S7. Final cryoEM processing flow chart for merged data from both datasets. Overview of the cryoEM processing steps for the combined datasets. Particles were picked with a Topaz model trained with the “good particles” from the final model for dataset 2. Mask for focused classification with partial signal subtraction around two helices and a region of DNA is shown in blue. Red oval highlights the area of the structure where resolution was improved after focused 3D classification.

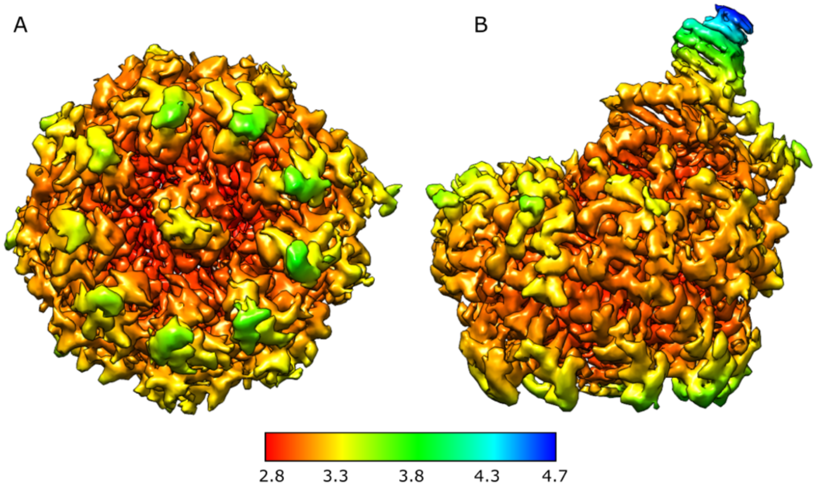

Fig. S8. CryoEM map coloured by local resolution. (**A**) Bottom view. (**B**) Side view.

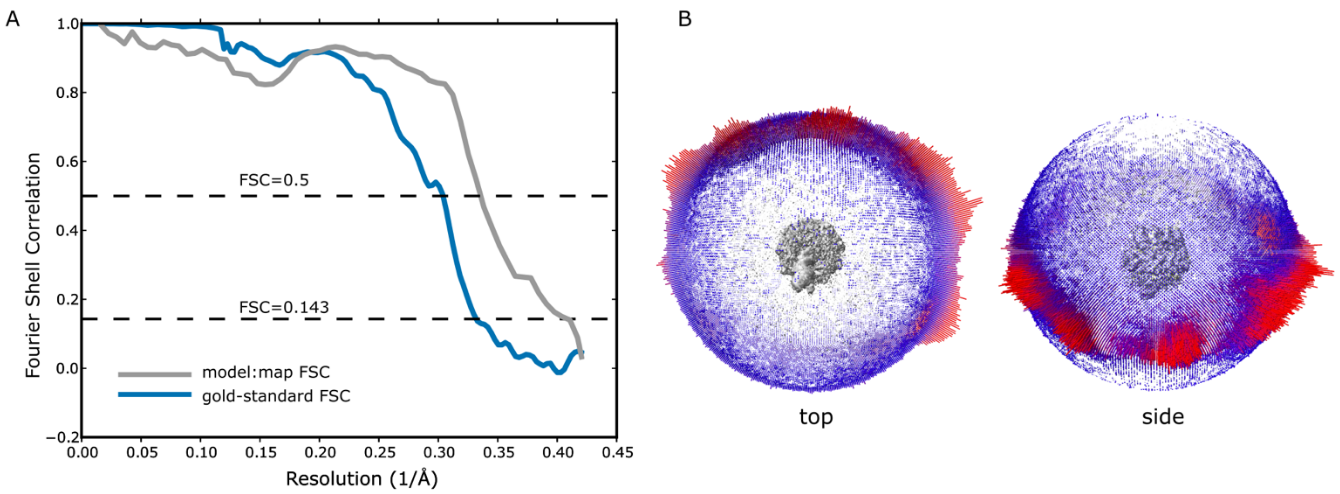

Fig. S9. (**A**) 'Gold standard' FSC curve (blue) and model:map FSC curve (grey). Dashed lines indicate FSC cut-offs of 0.143 and 0.5 for 'gold standard' and model:map FSC, respectively. (**B**) Euler angle distribution of particles contributing to the cryoEM reconstruction.

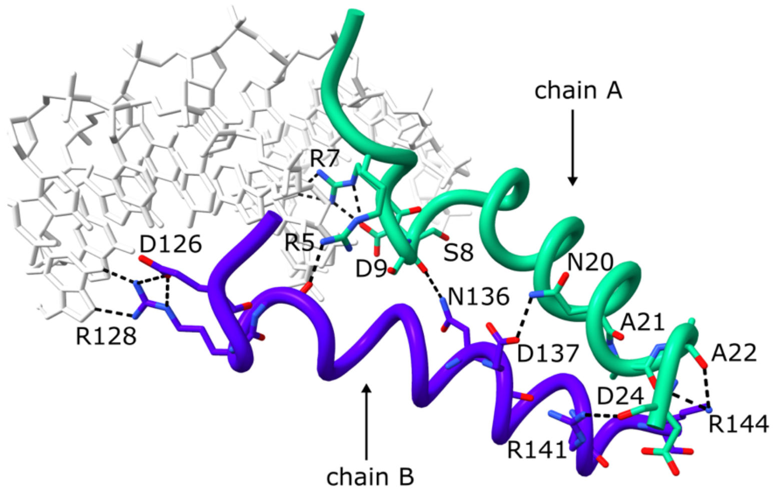

Fig. S10. DNA-binding substructure. N-terminus of chain A is shown in green, C-terminus of chain B is in purple, DNA is in white, hydrogen bonds are depicted as black dashed lines.

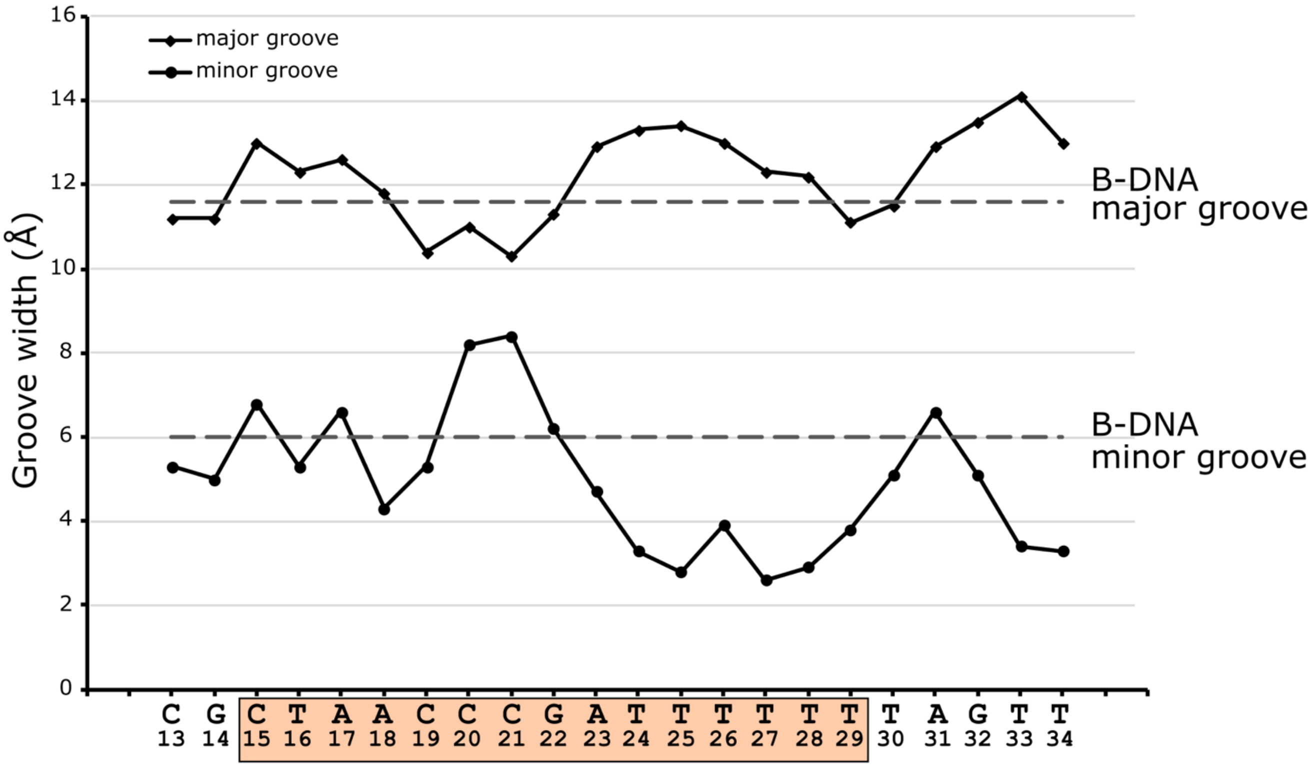

Fig. S11. Major and minor groove width. The widths of major and minor groove in DNA in the small terminase:DNA complex calculated using the program CURVES+ (*5*). The average major and minor groove widths of B-form DNA (*6*) are shown as a dotted line. Small terminase binding site is highlighted in orange.

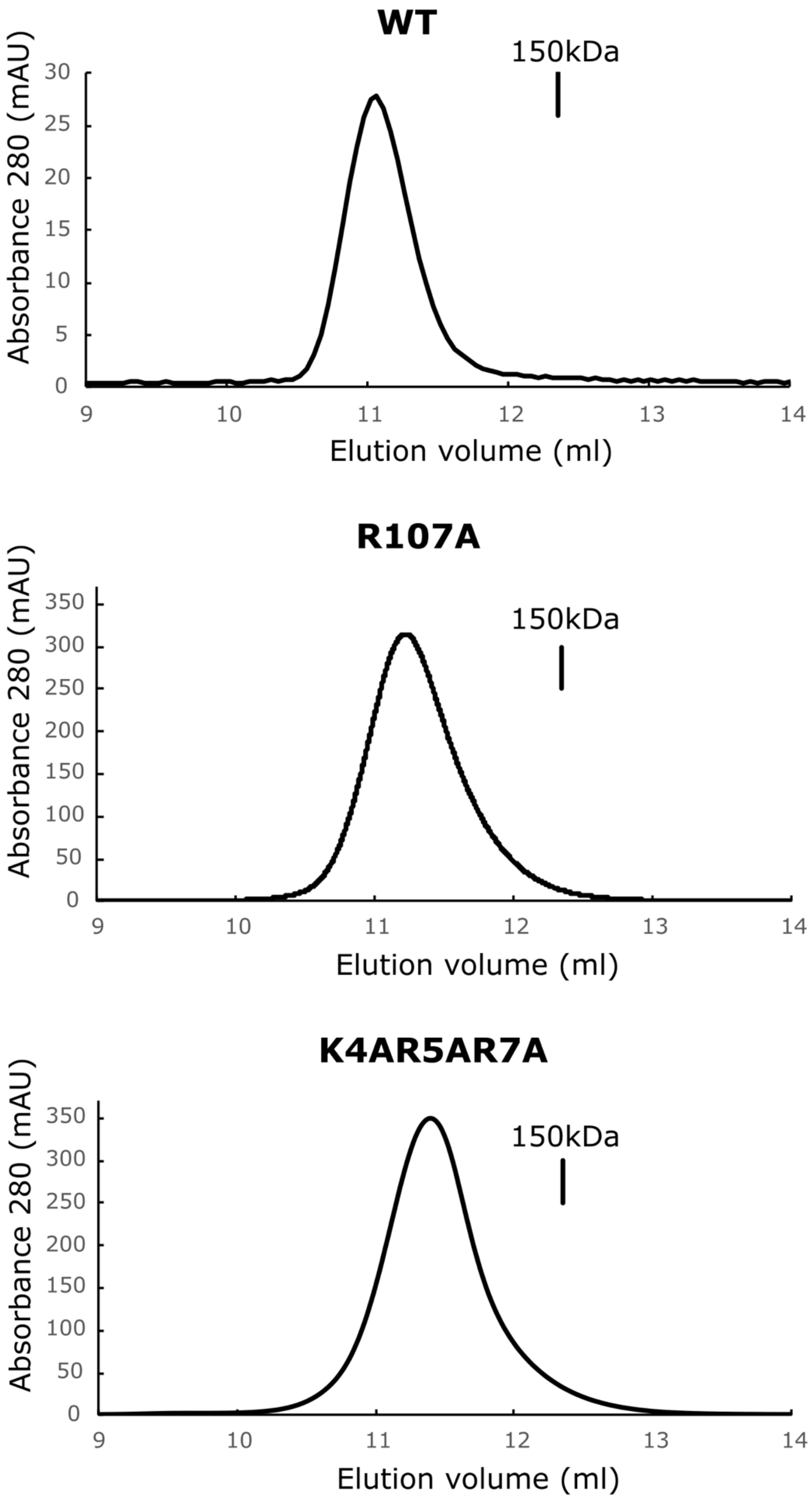

Fig. S12. Purification of small terminase mutants. Elution profiles of wild type (WT) protein and mutants R107A and K4AR5AR7A on S200 10/30 column.

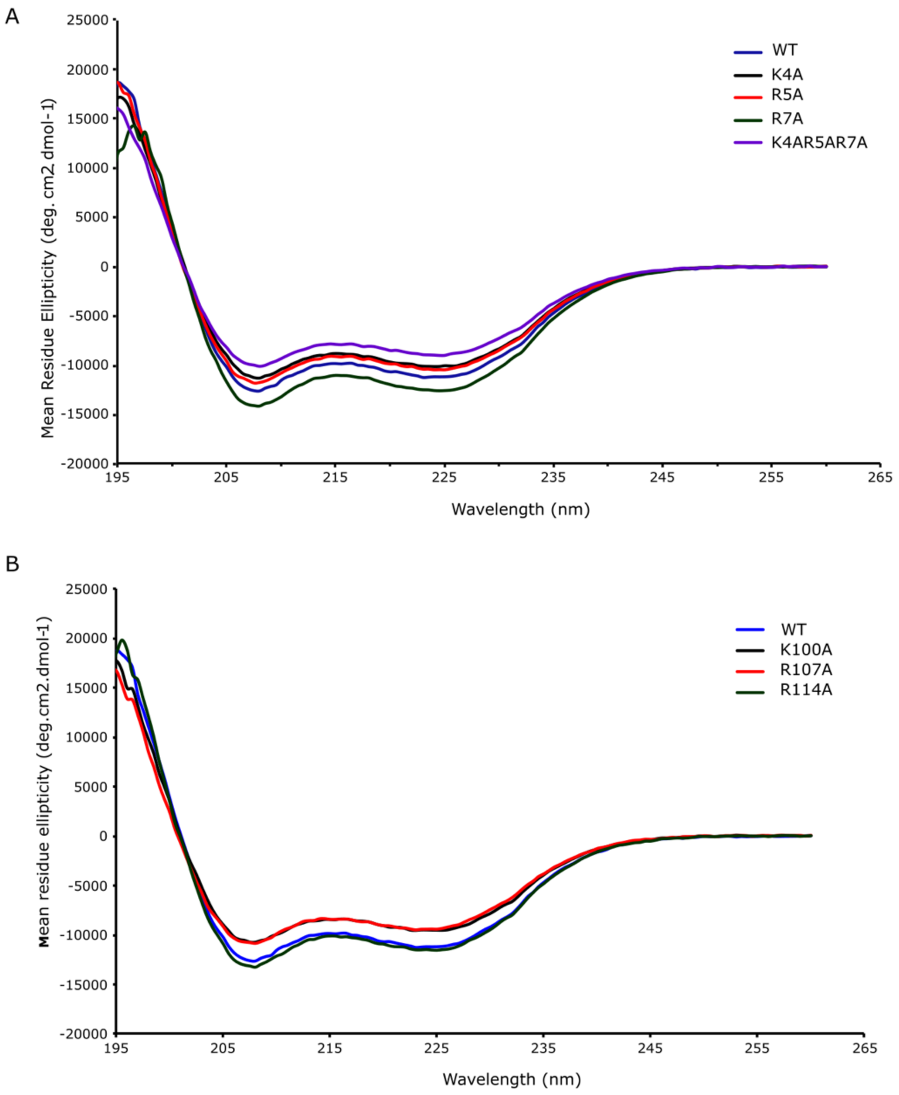

Fig. S13. Far-UV circular dichroism spectra of wild-type (WT) and mutants of small terminase. (**A**) Mutants of N-terminal arm. (**B**) Mutants within the channel of small terminase.

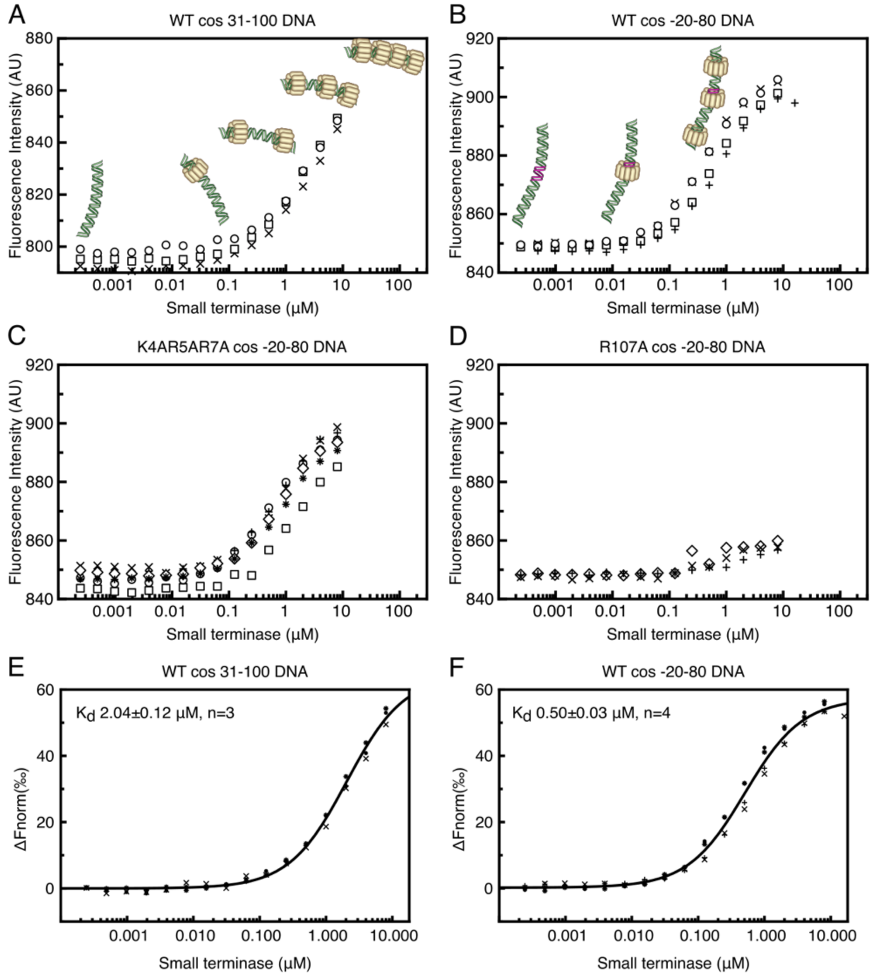

Fig. S14. MST binding curves. (**A**) Titration of cos 31-100 DNA, lacking the specific small terminase binding site, with wild type (WT) small terminase. (**B**) Titration of cos -20-80 DNA, containing the specific DNA binding sequence (magenta) with WT small terminase. (**C**) Titration of cos -20-80 DNA with K4AR5AR7A mutant of small terminase. (**D**) Titration of cos -20-80 DNA with R107A mutant of small terminase. (**E**) Fit of MST data from (**A**) with the standard single site equilibrium binding quadratic equation assuming a single protein binds to the DNA. (**F**) Fit of MST data from (**B**) with the standard single site equilibrium binding quadratic equation assuming a single protein binds to the DNA. Cartoons in panels A&B contain artwork from bioicons.org and reactome.org under CC BY 3.0 and CC BY 4.0 licences, respectively.

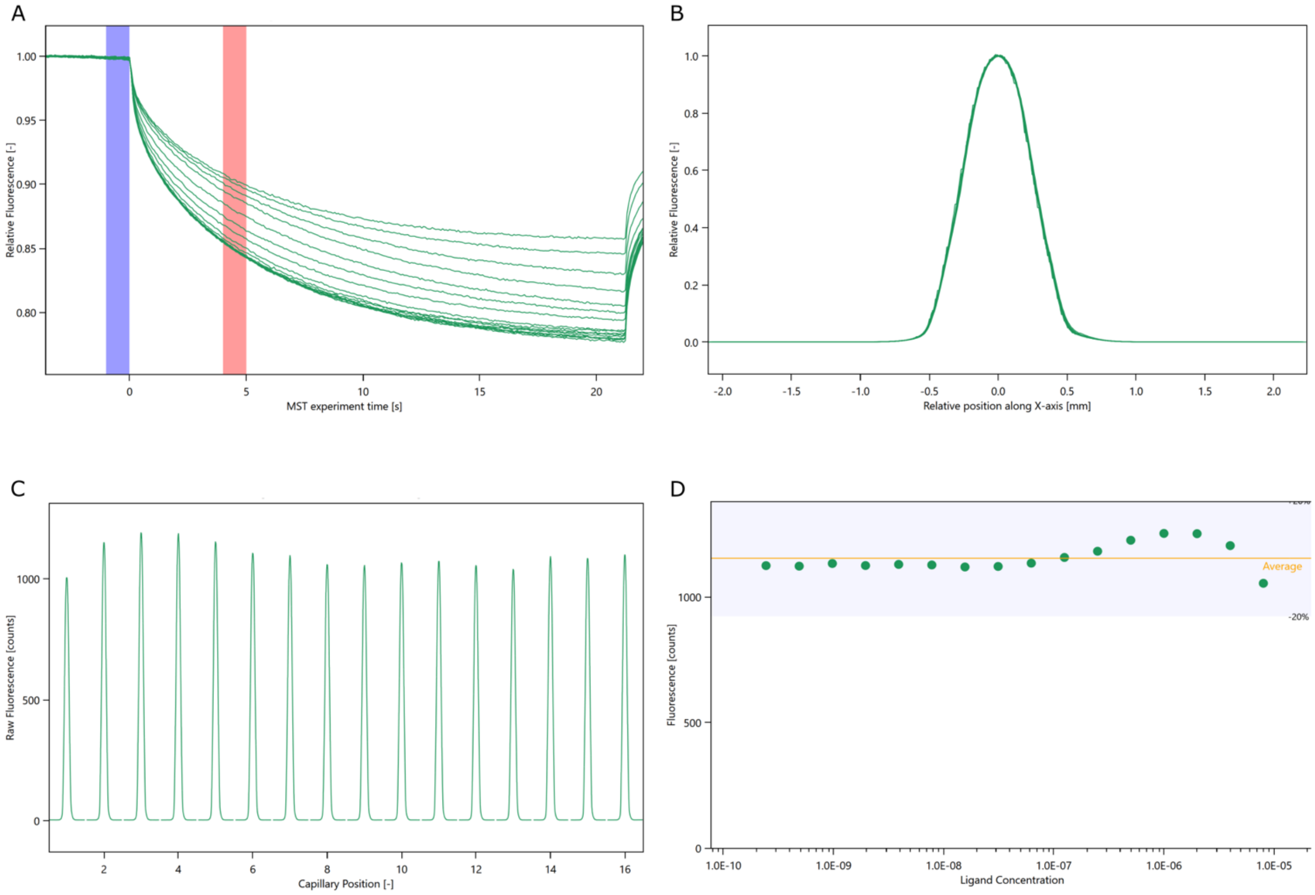

Fig. S15. MST original data for one run of WT small terminase. (**A**) Fluorescence traces over time for each capillary containing different concentrations of protein (**B**) Representative scan of fluorescence intensity across one capillary. (**C**) Representative capillary scan of fluorescent intensity across all capillaries. (**D**) Plot showing initial fluorescence intensity across all samples (capillaries) prior to temperature jump.

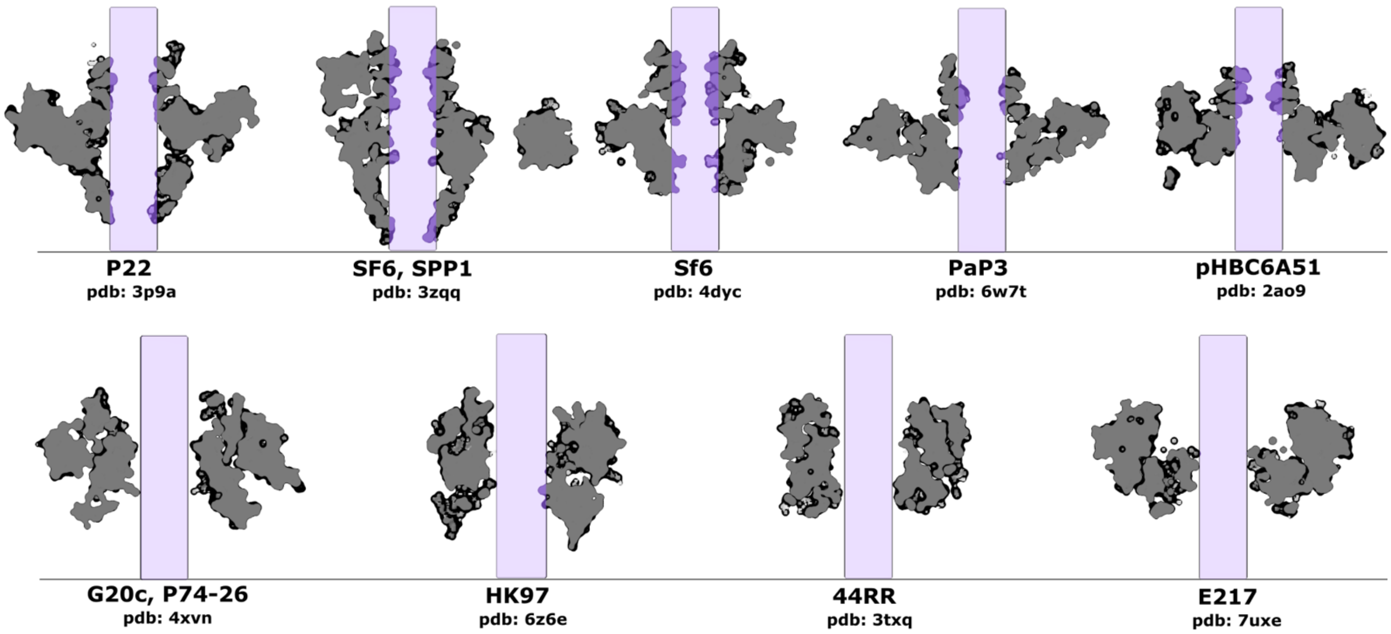

Fig. S16. Comparison of the central channel of small terminases. 3 Å thick section through the middle of small terminase models with 20 Å diameter cylinder positioned along the channel axis.

Table S1. Statistics for cryoEM data collection and processing.

Data collection	Dataset 1	Dataset 2
Microscope/detector	Krios/Gatan K2Summit with energy filter (slit width of 20 eV)	
Voltage (kV)	300	
Nominal magnification	130,000x	
Recording mode	counting	
Flux (e-/Å^2/s)	6.65	6.7
Target defocus (μm)	1.3 to 3.1	1 to 2.4
Pixel size (Å)	1.07	1.07
Fluence (e-/Å^2)	53	54
Number of fractions	50	50
Total exposure time (s)	8	8
Number of movies	682	2085
Total particles picked	306866	837519
Particles from both datasets used in final reconstruction	334433	
Map resolution at FSC = 0.143 (Å)	2.9	
Local resolution (Å)	2.9-4.9	
Map sharpening B-factor (Å^2)	-49.9	
Particles after focused classification with partial signal subtraction	76626	
Map resolution at FSC = 0.143 (Å)	3.0	
Map sharpening B factor (Å^2)	-30.9	

Table S2. Refinement statistics and validation.

Map resolution range refined against (Å)	3.0 – 114.1
Non-hydrogen atoms	
Protein	7784
Nucleic acid	1145
Average B factors ($Å^2$)	
Protein	87.5
Nucleic acid	115.5
R.m.s. deviations	
Bond lengths (Å)	0.0059
Angles (°)	1.335
Validation	
MolProbity score	1.11
Clashscore	3.16
Rotamer outliers (%)	0.12
Ramachandran plot	
Favoured (%)	99.25
Outliers (%)	0.0

Table S3. N-termini sequences of DNA binding proteins with N-terminal arm (NTA) and all known small terminases. Grey boxes - unmodelled residues, light blue boxes - NTA, red font - positively charged residues, yellow boxes - non structured residues, green boxes - helices, purple box - β-sheet.

PDB	Protein/phage	Sequence
5ZJQ	Homeobox extradenticle chain A	KKRKPYSKFQTLELEKEF
5ZJQ	Homeobox abdominal-B chain B	DARRKRRNFSKQASEILNEYFYS
1W0T	hTRF1	KRQAWLWEEDKNLRSGVRKYG
1W0U	hTRF2	KKQKWTVEESEWVKAG
HK97	Enterobacteria phage HK97	MADKRIRSDSSAAAVQAMKNAA
4Z3C	Bacillus phage SF6	MKEPKLSPKQERFIEEYFIN
3HEF	Enterobacteria phage Sf6	MATEPKAGRPSDYMPEVADDICSLLSS
3P9A	Enterobacteria P22	MAAPKGNRFWEARSSHGRNPKFESPEALWAAC
2A09	Bacillus cereus	MPFSISGRKGSEMMAKLDELKQKLTAK
3TXQ	44RR	MNDVLDFTQLKDLNGIEGIHGEDVQVYAPLVLRDPVSNPNNRKIDQDDDYELVRRN
4XNV	Thermus phage G20c	MSVSFRDRVLKLYLLGF
6W7T	Pseudomonas virus PaP3	MSDEKVVSIGAAPLSAKEKLDLYCE
7UXE	Pseudomonas E217	MTKFYSPDDLVTPQEFADPHFAAINQKRFDLYIDLRVQG
1J9I	Escherichia phage lambda	MEVNKKQLADIF

Table S4. Borders of DNA binding domains or N-terminus regions highlighted in gold in Fig. S1.

Phage	PDB	
P22	3p9a	N-terminus: 4-24
SP6	3zqq	HTH: 10-60
Sf6	4dyc	HTH: 10-52
PaP3	6w7t	HTH: 13-46
pHBC6A51	2ao9	HTH: 23-48
G20c	4xvn	HTH: 1-52
HK97	6z6e	-
44RR	3txq	N-terminus: 25-40
E217	7uxe	HTH: 14-50

Dataset S1 (separate file). Raw data for Fig. S14.

SI References

1. J. D. McGhee, P. H. von Hippel, Theoretical aspects of DNA-protein interactions: co-operative and non-co-operative binding of large ligands to a one-dimensional homogeneous lattice. *J Mol Biol* **86**, 469-489 (1974).

2. S. C. Kowalczykowski et al., Cooperative and noncooperative binding of protein ligands to nucleic acid lattices: experimental approaches to the determination of thermodynamic parameters. *Biochemistry* **25**, 1226-1240 (1986).

3. O. V. Tsodikov, J. A. Holbrook, I. A. Shkel, M. T. Record, Jr., Analytic binding isotherms describing competitive interactions of a protein ligand with specific and nonspecific sites on the same DNA oligomer. Biophys J **81**, 1960-1969 (2001).

4. L. M. Hellman, M. G. Fried, Electrophoretic mobility shift assay (EMSA) for detecting protein-nucleic acid interactions. *Nat Protoc* **2**, 1849-1861 (2007).

5. R. Lavery, H. Sklenar, Defining the structure of irregular nucleic acids: conventions and principles. *J Biomol Struct Dyn* **6**, 655-667 (1989).

6. R. Chandrasekaran, S. Arnott, The structure of B-DNA in oriented fibers. *J Biomol Struct Dyn* **13**, 1015-1027 (1996).
